# Acquisition and loss of CTX-M plasmids in *Shigella* species associated with MSM transmission in the UK

**DOI:** 10.1099/mgen.0.000644

**Published:** 2021-08-24

**Authors:** Rebecca K. Locke, David R. Greig, Claire Jenkins, Tim J. Dallman, Lauren A. Cowley

**Affiliations:** ^1^​ University of Bath, Claverton Down Campus, Bath, UK; ^2^​ Gastrointestinal Reference Services, Public Health England, London, UK; ^3^​ Division of Infection and Immunity, The Roslin Institute and Royal (Dick) School of Veterinary Studies, University of Edinburgh, Easter Bush, EH25 9RG, UK

**Keywords:** antimicrobial resistance, CTX-M, ESBL, MSM, public health, *Shigella*

## Abstract

Shigellosis in men who have sex with men (MSM) is caused by multidrug resistant Shigellae, exhibiting resistance to antimicrobials including azithromycin, ciprofloxacin and more recently the third-generation cephalosporins. We sequenced four *bla*_CTX-M-27_-positive MSM *Shigella* isolates (2018–20) using Oxford Nanopore Technologies; three *S. sonnei* (identified as two MSM clade 2, one MSM clade 5) and one *S. flexneri* 3a, to explore AMR context. All *S. sonnei* isolates harboured Tn7/Int2 chromosomal integrons, whereas *S. flexneri* 3a contained the *Shigella* Resistance Locus. All strains harboured IncFII pKSR100-like plasmids (67-83kbp); where present *bla*_CTX-M-27_ was located on these plasmids flanked by IS*26* and IS*903B*, however *bla*_CTX-M-27_ was lost in *S. flexneri* 3a during storage between Illumina and Nanopore sequencing. IncFII AMR regions were mosaic and likely reorganised by IS*26*; three of the four plasmids contained azithromycin-resistance genes *erm(B*) and *mph(A*) and one harboured the pKSR100 integron. Additionally, all *S. sonnei* isolates possessed a large IncB/O/K/Z plasmid, two of which carried *aph(3’)-Ib/aph(6)-Id/sul2* and *tet(A*). Monitoring the transmission of mobile genetic elements with co-located AMR determinants is necessary to inform empirical treatment guidance and clinical management of MSM-associated shigellosis.

## Data Summary

Whole genomes sequencing projects for strains 598 080 and 607 387 are deposited in GenBank under the accession numbers JAENSM000000000 and JAEMEC000000000 respectively. For strain 888 048, the chromosomal sequence can be retrieved with the accession CP066809 and its plasmids under accessions MW396860-3. For strain 893 916, the chromosomal sequence is deposited under accession CP066810 and plasmid sequences under accessions MW396858-9 and MW396864. Further information can be found in Table S4 (available in the online version of this article).

**Impact Statement**

Outbreaks of *Shigella *, a gastrointestinal pathogen, have been known to occur in men who have sex with men (MSM) globally since the 1970s. Increasing prevalence of multidrug resistance in Shigellae is concerning due to the challenge this poses to current antimicrobial treatment options. This includes the emergence of extended-spectrum beta-lactamase (ESBL)-producing strains which are resistant to ceftriaxone due to plasmid-mediated CTX-M determinants. As few CTX-M plasmids have been described, we apply a combination of short- and long-read sequencing with Illumina and MinION technologies to assemble complete plasmids in four *Shigella* isolates associated with MSM transmission in the United Kingdom to investigate the location of antimicrobial resistance (AMR) genes. We find *bla*_CTX-M-27_ is inserted into plasmids with high identity to known plasmids which have previously driven shigellosis epidemics worldwide and typically carry azithromycin resistance elements. We compare isolate differences in AMR gene context, the impact of insertion sequences on their plasmids and discuss how resistance profiles relate to MSM clade. Our findings highlight the necessity of increased public health monitoring of AMR in *Shigella* at different hierarchies; the whole genome, mobile genetic element and single gene level to understand resistance dissemination and inform effective treatment options.

## Introduction

*Shigella* is a Gram-negative genus comprising four species; *Shigella dysenteriae *, *Shigella boydii *, *Shigella sonnei* and *Shigella flexneri,* all etiological agents of shigellosis [1]. *Shigella* spp. are highly contagious [2] and primarily transmitted via the faecal-oral route through direct person-person contact or contaminated food or water [3]. Presence of a large virulence plasmid (pINV) encoding a type III secretion system supports *Shigella* invasion of colonic epithelial cells, generation of localised inflammation and necrosis, which combined with expression of genes on chromosomal pathogenicity islands (PAIs) generates characteristic symptoms of abdominal cramps and mucoid diarrhoea [4, 5]. Shigellosis accounts for a significant proportion of the global diarrhoea burden; despite mortality decreases since the 1990s, ~125 m estimated cases occur annually, mainly in children <5 years in Asia and Africa [6, 7].

Historically, *Shigella* infections in higher-income countries such as the United Kingdom (UK) were mainly associated with travellers returning from endemic areas [8]. However, since 2009 the UK has observed sustained epidemics of domestically-acquired multidrug resistant (MDR) *Shigella* serotypes *S. flexneri* 3a and 2a and *S. sonnei,* attributed to transmission in men who have sex with men (MSM) [9]. Ciprofloxacin, azithromycin, and ceftriaxone are now first and second-line treatment options [10] due to chromosomal acquisition of antimicrobial resistance genes (ARGs) relevant to earlier drugs. This includes the class II integron Tn7/Int2 (trimethoprim and aminoglycosides), class I *Shigella* Resistance Locus (SRL) (chloramphenicol, ampicillin and tetracycline) and additional sulphonamide resistance via the small spA plasmid [11–13]. However, ciprofloxacin use is restricted by step-wise chromosomal mutations in the quinolone resistance determining regions (QRDR) *gyrA* and *parC* [14] and intercontinental dissemination of azithromycin ARGs on a single large IncF plasmid, pKSR100, has directly enhanced previous MSM *Shigella* outbreaks [15].

Extended spectrum β-lactamase (ESBL) genes have implications for failure of ceftriaxone, one of the few remaining options, and were relatively rare in domestically-acquired *Shigella* in the UK [8]. However, in 2015 Public Health England (PHE) described an ESBL-producing *S. sonnei* MSM cluster [16] conferred by a pKSR100-like plasmid (p183660), which had acquired *bla*_CTX-M-27_. This element recently drove a prolonged *S. sonnei* outbreak among MSM in Victoria, Australia [17] but few CTX-M *Shigella* plasmids have been characterised [18]; seemingly only one other has been described in detail, in a Swiss *S. sonnei* isolate [19].

Assembly of these plasmids is important for AMR surveillance but is undermined by the failure of short-read sequencing to span repetitive mobile genetic element (MGE) regions, preventing ARG location determination [20]. Advent of long-read sequencing such as the Oxford Nanopore Technologies (Nanopore) platform, in combination with Illumina-mediated polishing, overcomes this to provide accurate genomes with complete plasmids [21, 22].

In this study we report comparative genomics of four MSM-associated *Shigella* strains (three *S. sonnei,* one *S. flexneri* 3a) in England initially characterised as *bla*_CTX-M-27_-positive. Availability of complete ESBL-producing *Shigella* genomes helps determine structural MGE arrangement, genomic ARG context and identify insertion sequences (IS) involved in their reorganisation. Together this helps elucidate how MGE evolution is tied to pathogenic persistence, emphasising the importance of responsible antimicrobial stewardship.

## Experimental procedures

### Short-read sequencing (Illumina HiSeq 2500) and data processing

Four strains of *Shigella* spp. (* Shigella sonnei *, *n*=3, *Shigella flexneri* 3a, *n*=1) from MSM were selected as representatives of clusters of all *Shigella* species in the PHE database with the *bla*_CTX-M-27_ determinant and characterised in this study (Table 1). If the isolate formed part of a cluster, the earliest isolate was selected with respect to faecal sample collection date (or date of isolation if sample data were unavailable). For Illumina sequencing, Genomic DNA was extracted from cultures using the QIAsymphony system (Qiagen). The sequencing library was prepared using the Nextera XP kit (Illumina) for sequencing on the HiSeq 2500 instrument (Illumina), run with the fast protocol. FASTQ reads were processed using Trimmomatic v0.27 [23] to remove bases with a PHRED score of <30 from the leading and trailing ends, with reads <50 bp after quality trimming discarded.

**Table 1.** Epidemiological information related to the four *Shigella* isolates associated with men who have sex with men (MSM) that were whole genome sequenced with Nanopore and Illumina technologies in this study. *S. sonnei* MSM clade is reported according to [8] and *S. sonnei* lineage is included according to [47]

Isolate ID	598 080	607 387	888 048	893 916
**Species**	* S. sonnei *	* S. sonnei *	* S. flexneri* 3a	* S. sonnei *
**Region**	London	London	London	London
**Sex**	M	M	M	M
**Age**	22	50	50	36
**Receipt date (M/Y**)	08/18	09/18	02/20	02/20
*** S. sonnei* MSM clade**	2	2	–	5
*** S. sonnei* lineage**	3.7.29.1.4.1 (Global III VN2.KH1.Aus)	3.7.29.1.4.1 (Global III VN2.KH1.Aus)	–	3.6.1.1.2 (CipR.MSM5)

### Whole genome long-read sequencing (Nanopore) and data processing

Samples were subsequently sequenced using Oxford Nanopore technologies. Genomic DNA was extracted and purified using the Fire Monkey DNA extraction kit (Revolugen) with subtle modifications to manufacturer’s instructions including removal of vortexing steps. Genomic DNA for each extract was quantified using a Qubit and the HS (high sensitivity) dsDNA assay kit (Thermofisher Scientific), following manufacturer’s instructions. Library preparation was performed using the Rapid barcoding kit SQK-RBK004 (Oxford Nanopore Technologies). The prepared libraries were loaded onto a FLO-MIN106 R9.4.1 flow cell (Oxford Nanopore Technologies) and sequenced using the MinION (Oxford Nanopore Technologies) for 36 h and further processed as follows (Fig. S1). Data produced in a raw FAST5 format was basecalled and de-multiplexed with Guppy v3.2.10 using the FAST protocol (Oxford Nanopore Technologies) into FASTQ format and de-multiplexed into each samples’ respective barcode.

Read metrics prior to and following filtering were determined with NanoPlot v1.8.1 [24]. Adapter sequences were trimmed from raw Nanopore reads and chimeric reads discarded to prevent cross-barcode contamination using Porechop v0.2.4 (https://github.com/rrwick/Porechop). Read filtering was conducted with Filtlong v0.2.0 (https://github.com/rrwick/Filtlong) to yield 30× theoretical coverage of the *Shigella* genome (~4.7 Mb) with the longest reads using the following parameters; min_length=1000, keep_percent=90, length_weight=10, target_bases=141 Mb.

### *De novo* assembly, polishing and reorientation

All genomes were *de novo* assembled from filtered ONT FASTQ files and two assemblers were compared for each strain. First, long-read assembly was performed with Flye v2.7.1 [25] with an input genome size of 4.7 Mb and use of the --plasmids parameter to rescue any small unassembled replicons. A hybrid assembly combining paired-end Illumina and Nanopore reads was also generated with Unicycler v0.4.8 [26]. The resulting assemblies were compared for contiguity (total contig number and contig N_50_) and plasmid recovery and the Flye-generated assembly was utilised for further investigation in all cases (Table S2). Assemblies were then polished to reduce complex break, insertion and base-level substitution errors; Illumina FASTQ reads were mapped to the draft assembly with the Burrows-Wheeler Aligner (BWA) v0.7.17 MEM algorithm [27] and Samtools v1.7 [28] with the -F 256 parameter to ignore non-primary aligning reads. This was achieved with two rounds of the Pilon v1.23 –fix all function [29] with the following parameters; minimum depth 0.05, minimum quality 30 and minimum mapping quality 30. This was followed by two rounds of Racon v1.4.13 [30] with a match score of 8, average base quality threshold for windows of 30, mismatch score of −6 and gap penalty of −8. The final assembly quality was determined by QUAST v5.0.2 [31]. Where possible, contigs were reoriented on *dnaA* or *repA* using the Circlator v1.5.5 fixstart function [32]. As one assembly (607387) was poorly resolved, contigs were re-ordered according to the high quality *S. sonnei* 53G reference (GenBank NC_016822.1) using Mauve v2.4.0 [33].

### Annotation, MLST and plasmid comparison

FASTA files were annotated with Prokka v1.14.6 [34] using a protein reference guide of pKSR100 (Accession LN624486) proteins and those identified at high confidence by ResFinder; in cases of ambiguity proteins were checked manually with the non-redundant blastp database. Then mlplasmids v1.1.0 [35] and blastn were used to predict chromosomal or plasmid contig origin. Multi-Locus Sequence Typing (MLST) was performed using the Centre for Genomic Epidemiology (CGE) database *E. coli* scheme #1 based on allelic profiles of seven housekeeping genes: *adk, fumC, gyrB, icd, mdh, purA* and *recA* [36]. IncFII plasmids were compared using blastn v2.10.1 with default parameters for percent identity and query cover to pKSR100 (GenBank LN624486.1), a conjugative plasmid from an *S. flexneri* strain SF795513 associated with shigellosis in MSM. As isolates were known to harbour *bla*_CTX-M-27_ they were also compared to p183660, found previously in *S. sonnei *, with high identity to pKSR100 and harbouring *bla*_CTX-M-27_ (GenBank KX008967.1) [16]. Other plasmids were compared to known plasmids using the non-redundant blastn database. ISsaga [37] was used to annotate IS in pKSR100-like plasmids and to detect overall numbers in genomes.

### Antimicrobial resistance and virulence gene identification

The CGE PlasmidFinder-2.0 and pMLST-2.0 Enterobacteriaceae plasmid replicon database [38] was used to detect and type plasmids with default parameters (>95 % nucleotide identity threshold and >60 % query coverage). Acquired ARGs in Shigellae samples were detected *in silico* from assembled sequences using the ResFinder-3.2 [39] database including known *E. coli* chromosomal mutations, both with default 90 % identity and 80 % minimum length thresholds but all genes found and reported exhibited >98 % identity and >99 % coverage. The Comprehensive Antimicrobial Resistance Database (CARD) Resistance Gene Identifier tool [40] was also used to identify ARGs and mutations with default parameters (Perfect and Strict hits), as ResFinder does not generally include chromosome-specific genes. To investigate discrepancies in ARG identification, Illumina and Nanopore reads were mapped to *bla*_CTX-M-27_ from the CARD database using BWA-MEM v0.7.17 and minimap2 v2.17 [41] respectively with Samtools v1.7 where BAM files were visualised using Tablet v1.19.09.03 [42]. The ISEScan tool v.1.7.2.1 [43] was used to identify terminal inverted repeats associated with IS. Virulence factors were detected within assemblies using VirulenceFinder (https://cge.cbs.dtu.dk/services/VirulenceFinder) with default parameters.

### Maximum-likelihood phylogeny

To provide context for the four *Shigella* isolates, maximum-likelihood phylogenies were created for the *S. flexneri* 3a isolate and 49 other *S. flexneri* genomes (50 total) (Table S3), and the *S. sonnei* isolates with 198 other *S. sonnei* strains (201 total) from PHE’s *Shigella* genome collection isolated in England. Representative strains from relevant unique 250 single-linkage hierarchical clusters were randomly selected from different time frames and SnapperDB v0.2.6 (get_the_snsps) [44] was used to generate core variant multiple sequence alignments of 4415 bp and 11 096 bp for *S. sonnei* and *S. flexneri* respectively. SnapperDB stores reference-based single nucleotide polymorphism (SNP) variant calls relative to the reference genome, with each isolate given a SNP Address based on single-linkage hierarchical clustering which is a proxy for the genetic distances of isolates in the population. In detail, Illumina reads were aligned to a reference genome, consisting of the *S. sonnei* strain Ss46 (GenBank accession number NC_007384.1) and *S. flexneri* 2a strain 2457T (GenBank accession number AE014073.1) using BWA-MEM v0.7.12 as previously described [45]. Sequence alignment maps were sorted and indexed to produce a binary alignment map (BAM) file using Samtools v1.0.18. SNPs were identified using GATK v2.6.5 and only high-quality SNP (mapping quality [MQ] >30, minimum depth >10, variant ratio >0.9) positions were extracted. These polymorphic position alignments were used to infer maximum-likelihood phylogenetic trees using IQ-Tree v2.0.6 [46] with 1000 ultrafast bootstrap approximations. The general time reversible model plus ascertainment bias correction was used to correct branch length overestimation due to absence of constant sites.

### Lineage typing

Discriminatory, lineage specific SNPs were defined based on the phylogenetic lineage assignment in Baker *et al*. [8] and extracted directly from SnapperDB v0.2.5. To ensure greater utility to public health researchers, lineage typing of *S. sonnei* isolates (including publicly available AUSMDU00008333 (GenBank accession ERA1715196) and 183 660 (GenBank accession SRX1766927)) was also performed according to a newly described standard scheme [47] with Mykrobe v0.9.0 (predict) software [48] using the --ont parameter with trimmed FASTQ reads as input.

### Metadata and ARG presence for isolates used for phylogenetic context

Not all isolates included for context were MSM-linked, however shigellosis cases that were (i) male, (ii) adults and (iii) had not reported recent foreign travel were inferred to be likely associated with sexual transmission among MSM. Presence of genes conferring resistance to azithromycin (*erm(B), mph(A*)) and mutations in the quinolone resistance-determining regions (QRDR) of *gyrA* and *parC* for all isolates included in the two phylogenetic trees were extracted from a previously published study [45]. This excludes *S. sonnei* isolate 893 916 where ResFinder results were used to determine presence of these genes and mutations. Detection of *bla*_CTX-M-27_ within all genomes was determined using a mapping-based approach known as GeneFinder v2.2 (https://github.com/phe-bioinformatics/gene_finder) with default parameters. GeneFinder utilises Bowtie2 v2.1 [49] and Samtools v1.0.18 [28] to align Illumina query sample reads to a reference.1.1.

### Visualisation tools

Graphical comparison of chromosomal islands was performed using EasyFig v2.2 [50] and SRL genomic features of *S. flexneri* 3a were visualised with GView 1.7 [51]. blast Ring Image Generator (BRIG) [52] with blast v2.10.1 was utilised to visualise similarity of plasmids to pKSR100 and p183660, the latter of which was used as a reference. BRIG was also used to compare IncB/O/K/Z plasmids to a plasmid identified with high similarity by blastn, pAUSMDU00008333_3 (GenBank LR213460.1); isolate 607 387 was used as a reference due to it being the largest plasmid and virulence plasmids (pINV) were compared to one another other. The R package ggtree [53] was used to midpoint root maximum-likelihood trees, annotate tip nodes and visualise trees with isolate metadata and resistance gene presence/absence as an adjacent heatmap.

## Results

### Genomic features and assemblies

Four MSM-associated *Shigella* isolates (three *S. sonnei;* two 2018, one 2020 and one *S. flexneri* 3a, 2020) were selected for long-read sequencing due to *bla*_CTX-M-27_ presence in initial Illumina reads (Table 1). In all cases Flye outperformed Unicycler in creating more contiguous assemblies (Tables 2 and S2); detailed contig-level information for each isolate is available in Table S4.

**Table 2.** Assembly statistics, genomic features and predicted number of insertion sequence (IS) elements of the three *S. sonnei* isolates and one *S. flexneri* 3a isolate. Genomes were constructed with Flye, where Illumina short reads were utilised to polish a draft long-read assembly to yield a more complete and accurate final assembly

Isolate ID	598 080	607 387	888 048	893 916
**Species**	* S. sonnei *	* S. sonnei *	* S. flexneri* 3a	* S. sonnei *
**MLST**	152	152	245	152
**Total contigs**	6 (2 Chromosomal contigs, 3 plasmids, 1 likely plasmid contig)	37 (24 likely Chromosomal contigs, 4 plasmids, 9 likely plasmid contigs)	5 (1 Chromosomal contig, 2 plasmids, 2 likely plasmid contigs)	4 (1 Chromosomal contig, 3 plasmids)
**Total size (bp**)	5 282 682	5 315865	4 839 032	5 196 904
**Largest contig (bp**)	4 879 016	2 500 534	4 519 004	4 813 904
**N_50_**	4 879 016	897 518	4 519 004	4 813 904
**L_50_**	1	2	1	1
**GC content (%)**	50.86	50.82	50.68	50.84
**Total CDS**	5533	5554	5174	5462
**rRNA**	22	22	23	22
**tRNA**	102	101	98	96
**tmRNA**	1	1	1	1
**Acquired resistance genes (ResFinder**)	11	9	7	11*
**Resistance genes (CARD)†**	59	58	47	60
**Virulence factors (VFDB**)	14	13	12	13
**Predicted IS number**	577	520	504	588
**Estimated different IS**	45–48	38–43	43–47	43–44
**SRR number (Illumina**)	SRR7842065	SRR7892120	SRR11096691	SRR11206407
**SNP address**	1.3.197.460. 1360.1582. 2770	1.3.197.460. 1360.1582. 2813	35.43.43. 43.1189. 2224.2978	1.1.1.1.377. 394.3945

*Two mutations in *gyrA* are counted as two genes.

†Perfect and Strict hits only, total includes those also found by ResFinder.

### Virulence determinants and known virulence plasmid, pINV

Detection of virulence factors revealed small differences between strains and no genes encoding Shigatoxin (Table 3). All isolates harboured the large ~220 kbp virulence plasmid pINV (Fig. S5); pivotal for *Shigella* pathogenic evolution through acquisition of genes such as the conserved 30kbp *mxi-spa* locus encoding a type III secretion system, facilitating host invasion and spread [54]. pINV carried *ipaD*, *capU* and *virF*, with the *S. flexneri* isolate containing an additional *sepA* gene, thought to intensify intestinal fluid accumulation [55]. *S. sonnei* isolates displayed similar virulence profiles to *S. flexneri* but lacked *sat*, instead possessing *lpfA*, *sigA* and *senB*, hallmarks of *S. sonnei* virulence [56].

**Table 3.** Virulence profiles of four *Shigella* isolates sequenced in this study, as determined by VirulenceFinder from whole genome assemblies. x indicates virulence factor present, – indicates virulence factor absent

Virulence factor	598 080 (*S. sonnei)*	607 387 (*S. sonnei)*	888 048 (* S. flexneri* 3a)	893 916 (* S. sonnei *)
Invasion protein (*ipaD*)	x	x	x	x
Hexosyltransferase homlog (*capU*)	x	x	x	x
VirF transcriptional activator (*virF*)	x	x	x	x
* Shigella* extracellular protein A (*sepA*)	–	–	x	–
Glutamate decarboxylase (*gad*)	x	x	x	x
Invasion plasmid antigen (*ipaH*)	x	x	x	x
Aerobactin synthetase (*iucC*)	x	x	x	x
Ferric aerobactin receptor (*iutA*)	x	x	x	x
Secreted autotransporter toxin (*sat*)	–	–	x	–
Iron transport protein (*sitA*)	x	x	x	x
Tellurium ion resistance protein (*terC*)	x	x	x	x
Outer membrane complement resistance protein (*traT*)	x	x	x	x
Long polar fimbriae (*lpfA*)	x	x	–	x
* Shigella* IgA-like protease homolog (*sigA*)	x	x	–	x
Enterotoxin ShET-2 (*senB*)	x	x	–	x
Endonuclease colicin E2 (*celb*)	x	x	–	–

### Shared chromosomal AMR determinants among *Shigella* species

**Fig. 1.**
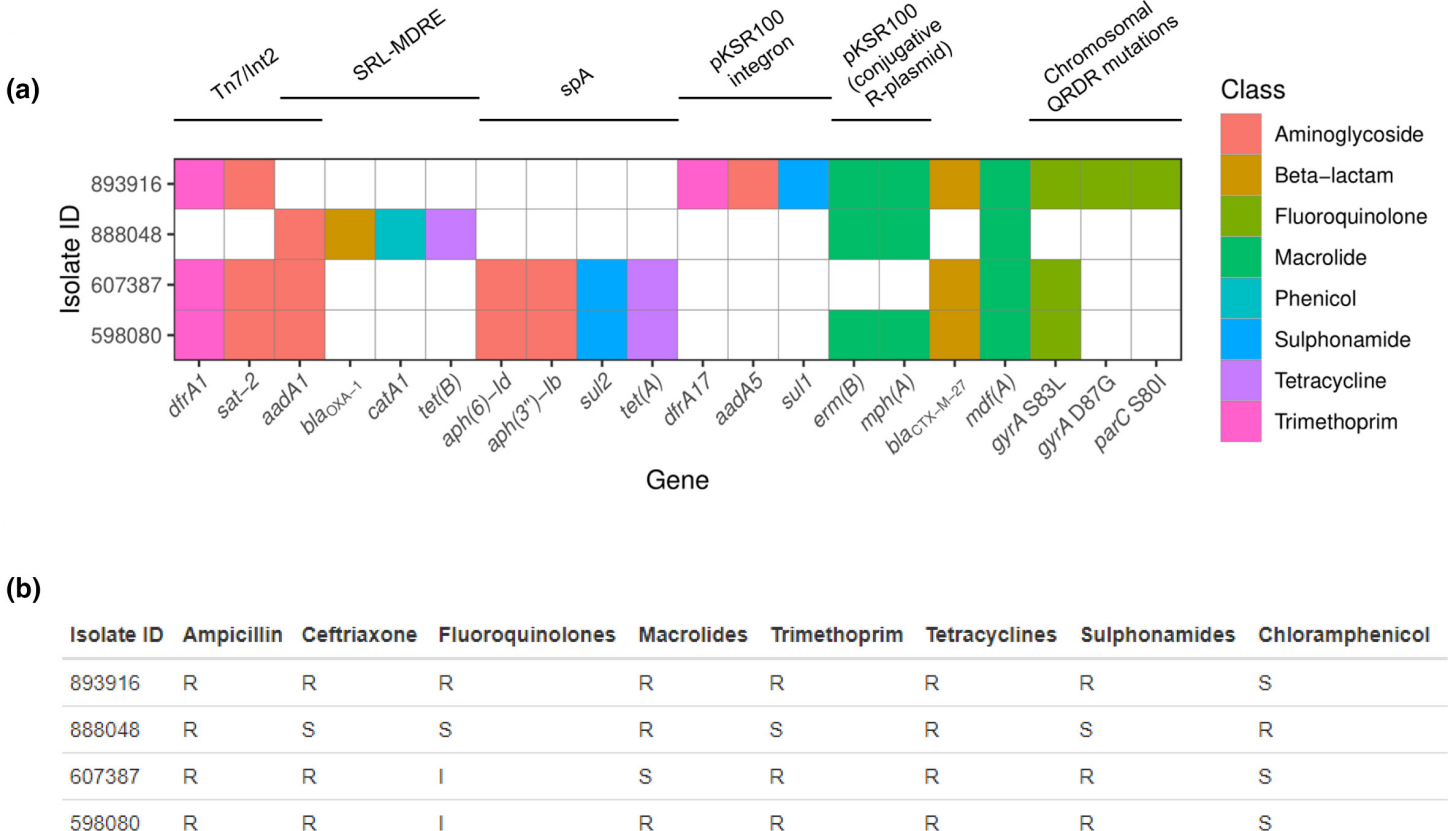
(**a**) Binary heatmap of antimicrobial resistance genes (ARGs) and chromosomal mutations present in four *Shigella* strains identified by ResFinder-3.2; *S. sonnei* (598 080, 607 387 and 893 916) and *S. flexneri* 3a (888048). Where a gene is present (at a threshold of >98 % identity and >99 % query coverage), the tile is coloured by the antimicrobial class it confers resistance to. The gene *sat-2* is not included in the ResFinder database but is included in this figure and chromosomal mutations in quinolone resistance determining region (QRDR) genes *gyrA* and *parC* are shown on the right. Known mobile genetic elements in *Shigella* are denoted above, as previously defined by Baker *et al*. [8]. (**b**) Phenotypic resistance profiles of the four isolates to antimicrobial classes. R=Resistant, S=Susceptible, I=Intermediate.

**Fig. 2.**
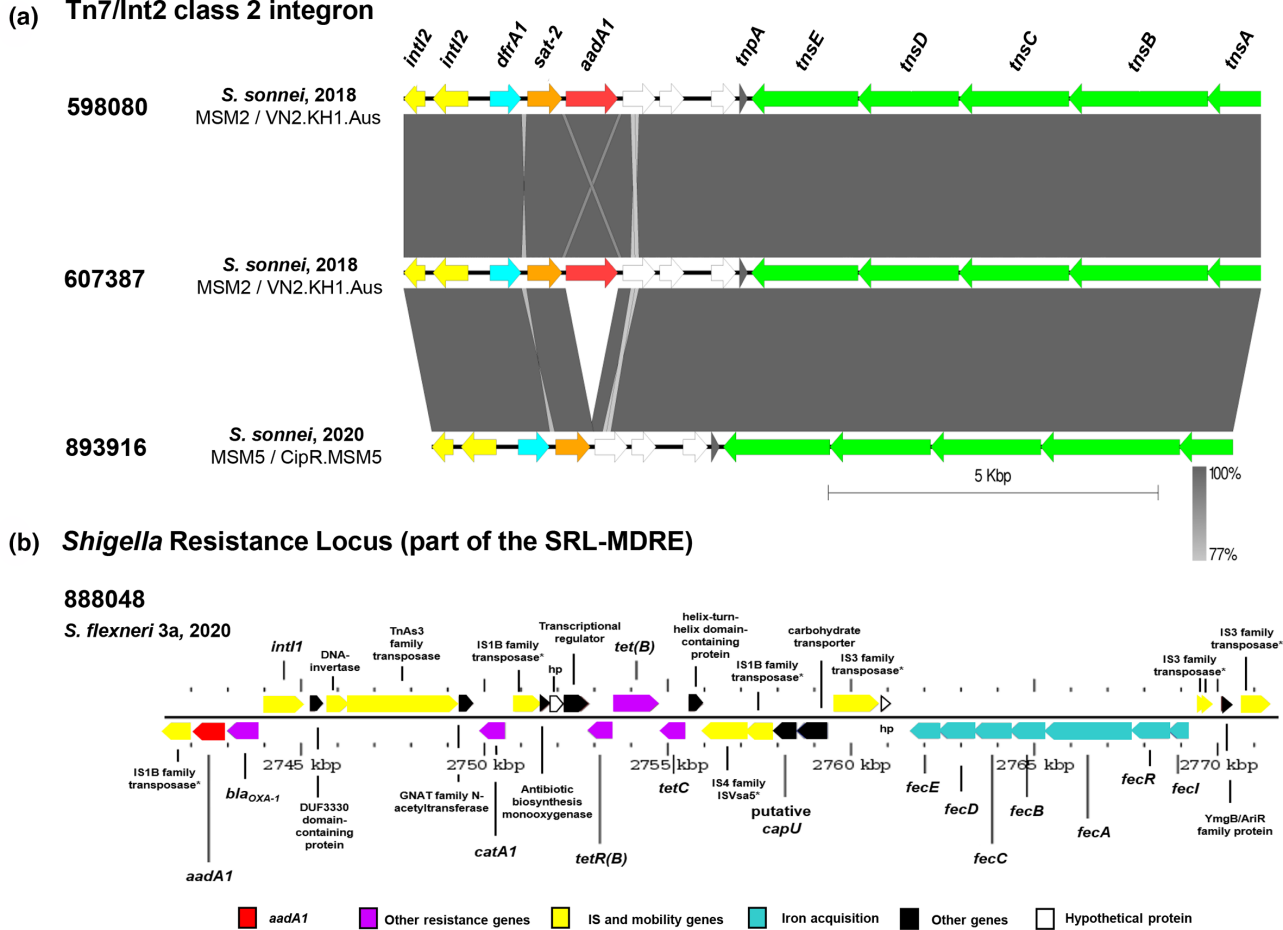
Chromosomal resistance islands identified in four MSM-associated *Shigella* isolates through sequencing with Oxford Nanopore Technologies. (**a**) blast comparisons of Tn7/Int2 integron antibiotic resistance gene cassettes found in all strains of *S. sonnei *. The three have similar arrangements, with 893 916 lacking the 3′ *aadA1* aminoglycoside resistance gene. *IntI2,* an integrase, is shown in yellow and is non-functional. Genes *dfrA1:* dihydrofolate reductase (blue); *sat-2:* streptothricin acetyltransferase (orange); *aadA1*; aminoglycoside adenyltransferase (red); encoding resistance to trimethoprim, streptothricin and streptomycin respectively. Hypothetical proteins are shown in white and Tn7 transposition genes shown in green. The direction of the arrow shows the direction of gene coding and grey gradient legend shows blast identity between sequences. Figure produced with Easyfig v2.2.5 [50]. (**b**) Genomic organisation of the chromosomal Shigella Resistance Locus, part of the Multiple Drug Resistance Element (SRL-MDRE) present only in the *S. flexneri* 3a strain 888 048. This harbours *aadA1, bla_OXA-1_, catA1* and *tet* genes encoding resistance to aminoglycosides, ampicillin, chloramphenicol and tetracycline respectively. *aadA1* is shown in red due to commonality to Tn7/Int2, other resistance genes are shown in purple. The iron acquisition system is shown in turquoise, mobility related genes in yellow, other genes in black and hypothetical proteins in white. Figure produced with Gview [51].

All isolates were genotypically and phenotypically MDR (resistant to ≥3 antimicrobial classes) and harboured known *Shigella* MGEs (Fig. 1) according to [8] (Table S5). Sequence analysis showed the presence of chromosomal class 2 IntI2/Tn*7* integrons in all *S. sonnei* isolates. For 598 080 and 607 387 this comprised the canonical ARG cassette organisation *dfrA1-sat2-aadA1* (Fig. 2a), conferring trimethoprim, streptothricin and streptomycin resistance respectively, however 893 916 lacked *aadA1*. This was unexpected as IntI2 is unable to alter the array due to an internal stop codon, usually leading to constant arrangements. All *S. sonnei* isolates harboured a 3′ *tns* transposition gene segment (*tnsA-E*) inserted adjacent to glutamine-fructose-6-phosphate aminotransferase, *glmS,* suggesting a single acquisition event. No such cassette was present in the *S. flexneri* isolate, which instead harboured *aadA1* on a class one integron, the SRL (Fig. 2b) alongside *bla*_OXA-1_ *, catA1* and *tet(B*) genes. These confer resistance to streptomycin, β-lactams, chloramphenicol and tetracyclines alongside a ferric uptake transport system (*fecI-R* with downstream structural *fecABCDE* genes), all integrated into tRNA-Ser.

Ciprofloxacin resistance is conferred by chromosomal Quinolone Resistance Determining Region (QRDR) mutations [8]. The two 2018 *S. sonnei* strains harboured single *gyrA* mutations (S83L) conferring reduced susceptibility to fluoroquinolones and are concerning as this can act as a prerequisite for successive stepwise mutations [57], implying possible evolution into fully resistant clones. Contrastingly, the 2020 *S. sonnei* strain had triple mutations (*gyrA* S83L; D87G, *parC* S80I) conferring high-level resistance. No QRDR mutations were found in *S. flexneri* 3a and, overall, no plasmid-encoded quinolone ARGs were detected.

All isolates harboured chromosomal class C β-lactamases *ampC* (between *frdD* and *blc* genes) and *ampH* (between *sbmA* and *iprA*) (Fig. 1b) promoting cephalosporin resistance [58]. These results are all concordant with phenotypic data, excluding the finding that 893 916 was phenotypically resistant to tetracyclines, despite no *tet* genes being found by ResFinder or CARD (Figs 1b and S2).

### Phylogenetic analysis and clustering within MSM clades

**Fig. 3.**
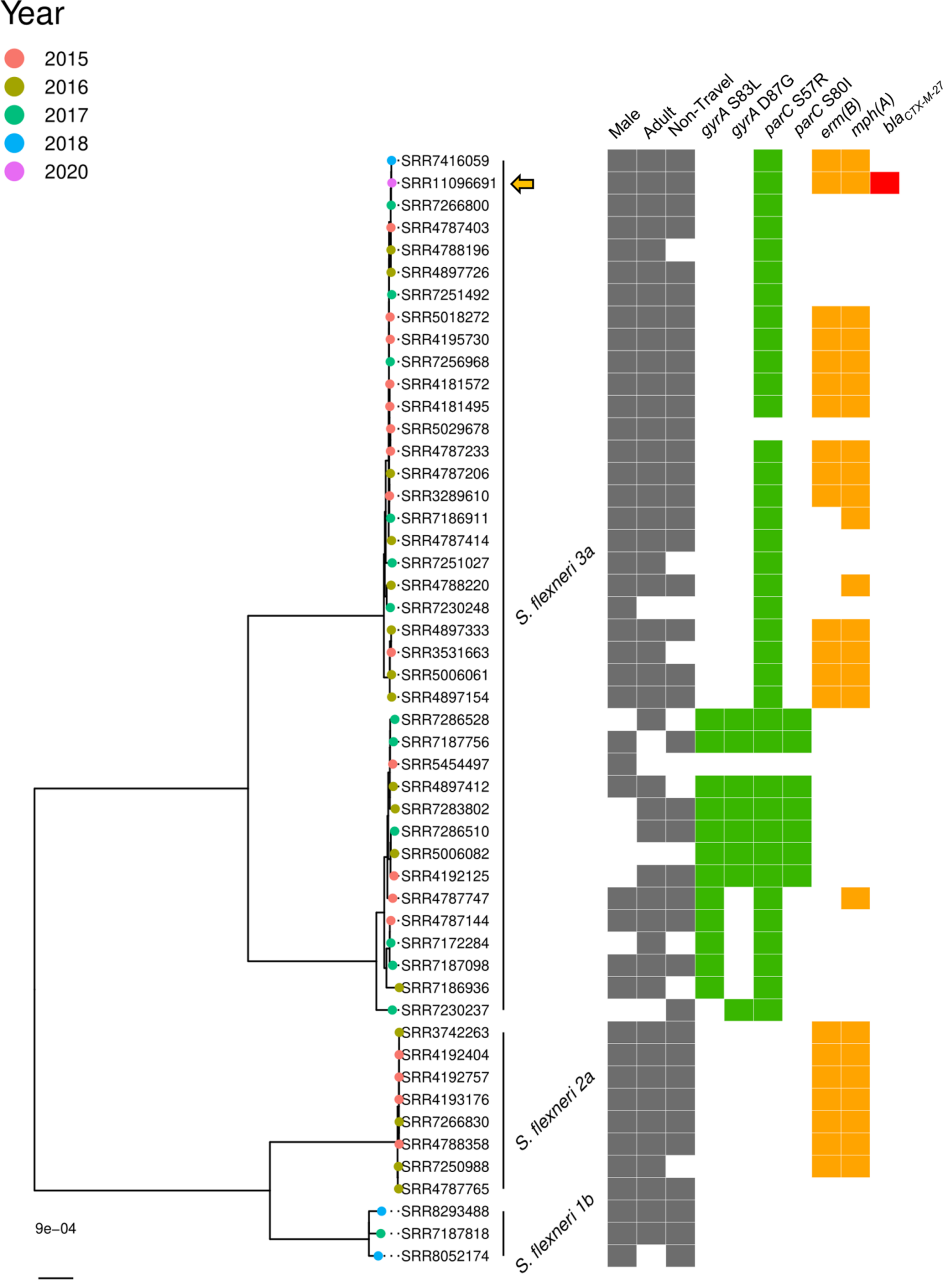
Maximum-likelihood phylogeny of *S. flexneri* 3a isolate 888 048 (SRR11096691, indicated by a filled orange arrow) sequenced with Nanopore technologies in this study, compared to other *S. flexneri* isolates in Public Health England’s collections. To place the isolate in context, 49 other *S. flexneri* isolates that are representatives from relevant SNP (single nucleotide polymorphism) clusters were included in the comparison. MSM clades associated with *S. flexneri* 1b, *S. flexneri* 2a and *S. flexneri* 3a are labelled. Isolates are labelled by SRR number and tip nodes are coloured by year of receipt. Adjacent grey blocks in each of the first three columns indicate the sample was isolated from a male, an adult, and that the illness was non-travel related (left-right). Grey blocks in these three lanes for an isolate implies the infection may be associated with MSM transmission. Presence of antimicrobial resistance genes concerning fluoroquinolones (mutations in *gyrA* and *parC*, green) and macrolides (*erm(B*) and *mph(A),* orange) are taken from Bardsley *et al*. [45] and presence of *bla_CTX-M-27_* was determined using GeneFinder. Gene presence is indicated by a coloured tile in the relevant column. ML trees are midpoint rooted and scale bar indicates SNPs.

To determine evolutionary relationships of strains within *Shigella *, we undertook separate analyses of 50 *S *. *flexneri* isolates and 201 *S *. *sonnei* strains based on SNP differences. The *S. flexneri* 3a isolate clustered within an *S. flexneri* 3a clade with high genetic homogeneity that emerged in 2015 and persists in the MSM population to date (Fig. 3), suggesting a sustained outbreak with possibly one transmission chain. Previous phylodynamic analysis has shown decreases in *S. flexneri* 3a cases and effective population size over time in English MSM, reflected in decreased sample diversity [45] as displayed here. Isolate 888 048 was the only *S. flexneri* isolate with presence of *bla*_CTX-M-27_, within a clade where almost all isolates harbour single *parC* mutation S57R and the majority contain both *erm(B*) and *mph(A*), though some isolates exhibit neither macrolide resistance determinant or *mph(A*) alone.

**Fig. 4.**
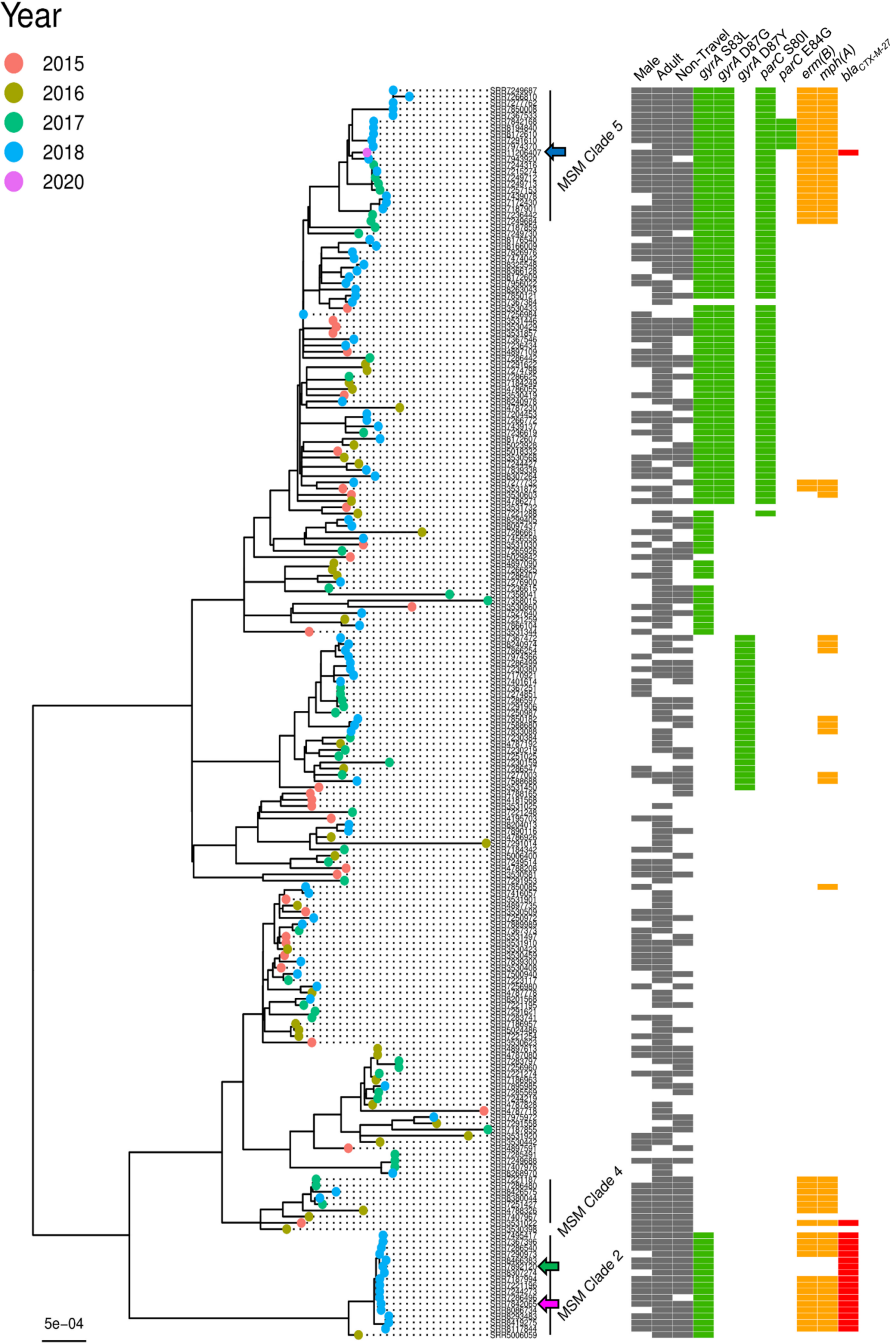
Maximum-likelihood phylogeny of three *S. sonnei* isolates known to be associated with men who have sex with men that were sequenced using Nanopore technologies in this study, compared to other isolates in Public Health England’s collections. To place the isolates in context, 198 other *S. flexneri* isolates that are representatives from relevant SNP (single nucleotide polymorphism) clusters were included in the comparison. *S. sonnei* isolates investigated in detail in this study are as follows; 598 080 (SRR7842065, pink filled arrow), 607 387 (SRR7892120, green filled arrow), 893 916 (SRR11206407, blue filled arrow). Isolates are labelled by SRR number and tip nodes are coloured by year of receipt. *S. sonnei* MSM clades are labelled as per those described in Baker *et al*. [8]. Adjacent grey blocks in each of the first three columns (left-right) indicate the sample was isolated from a male, an adult, and that the illness was non-travel related (left-right). Grey blocks in these three lanes for an isolate implies the infection may be associated with MSM transmission. Presence of antimicrobial resistance genes concerning fluoroquinolones (mutations in *gyrA* and *parC*, green) and macrolides (*erm(B*) and *mph(A),* orange) are taken from Bardsley *et al.* [45] and presence of *bla_CTX-M-27_* was determined using GeneFinder. Gene presence is indicated by a coloured tile in the relevant column. ML trees are midpoint rooted and scale bar indicates SNPs.

*S. sonnei* is divided into four lineages, one of which (lineage III) disseminated globally after MDR acquisition [11], and comprises five MSM-specific clades [8]. The two 2018 *S. sonnei* isolates (green and pink triangles) clustered within MSM clade 2 alongside mostly 2018 isolates (Fig. 4). This clade is known to have acquired pKSR100 (with *erm(B*) and *mph(A*)) on multiple occasions, have single QRDR mutations and links to Australian MSM but has a declining effective population with stable case rates [45]. Lineage typing according to a new scheme denotes 598 080 and 607 387 to be part of the VN2.KH1.Aus lineage (genotype 3.7.29.1.4.1); Australian isolates that emerged from the Khanh Hoa region of Vietnam [47].

Contrastingly, the 2020 isolate clustered within MSM clade 5 isolates collected from 2017 to 2020. Members are known to harbour pKSR100 and concerning triple QRDR mutations (some exhibited an additional *parC* E84G mutation) and have an increasing effective population size since 2017 [45]. When utilising the standard typing scheme, 893 916 was part of the CipR.MSM5 sublineage (genotype 3.6.1.1.2), which emerged from South Asia in the early 2000s, is linked to MSM communities and is responsible for the majority of fluoroquinolone resistant *S. sonnei* infections in Australia, England and the USA [47].

We found *bla*_CTX-M-27_ is not restricted to one *S. sonnei* MSM clade and may have been acquired on multiple occasions; 893 916 is the only MSM clade 5 isolate with this element whereas it is present in all MSM clade 2 and only one MSM clade 4 isolate (Fig. 4). *bla*_CTX-M-27_ is only found in isolates with both *erm(B*) and *mph(A*) (likely co-located on the same plasmid) or neither determinant. Clustering of non-MSM cases within MSM clades could indicate community acquisition from MSM (e.g. shared households), or onward transmission to MSM from travel-related cases.

### *S. sonnei* harbour AMR determinants on IncB/O/K/Z plasmids

**Fig. 5.**
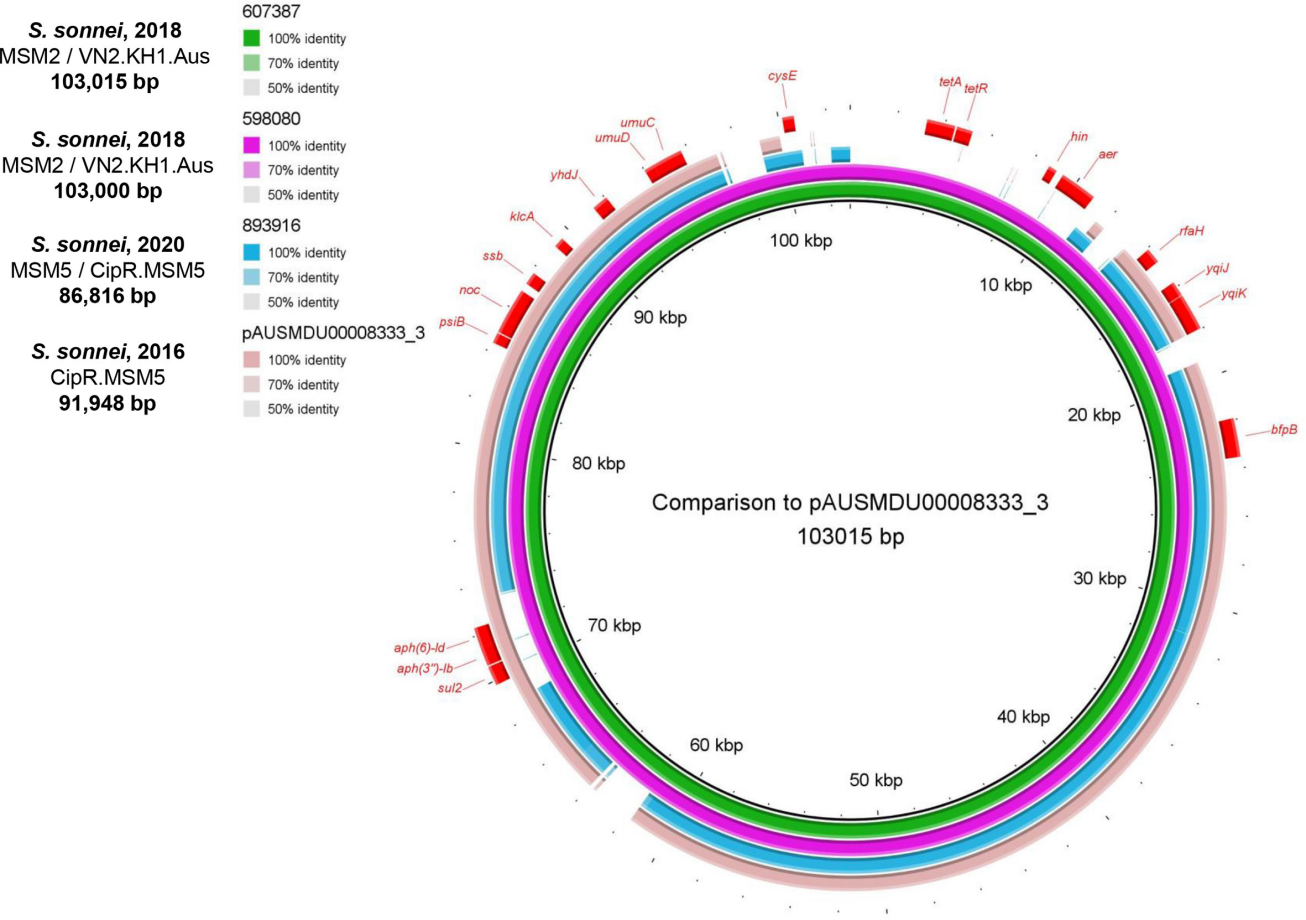
blast comparisons of IncB/O/K/Z plasmids for three MSM *S. sonnei* isolates in England, obtained with Nanopore sequencing. These are compared to a plasmid known to be associated with MSM in Australia, pAUSMDU00008333 plasmid 3 (LR213460.1). Three *S. sonnei* plasmids are from this study (isolates 598080; pink ring, 607387; green ring and 893916; blue ring) and the outer peach coloured ring is pAUSMDU00008333 plasmid 3. Genes present are shown in red on the outer ring (excluding hypothetical proteins). *aph(3′)-Ib (strA*): Aminoglycoside 3′-phosphotransferase*; aph(6)-Id* (*strB*): Aminoglycoside O-phosphotransferase; *sul2*: Dihydropteroate synthase; *tet(A):* Tetraycline efflux protein; *psiB:* Plasmid SOS inhibition protein B; *noc:* Nucleoid occlusion protein; *ssb:* Single-stranded DNA-binding protein; Anti-Restriction protein; *yhdJ:* DNA adenine methyltransferase*; umuD* and *umuC -* UV mutagenesis and repair proteins; *cysE:* Serine acetyltransferase; *hin:* DNA-invertase; *aer:* Aerotaxis receptor; *rfaH:* Transcription antitermination protein; *yqiJ* and *yqiK:* Inner membrane proteins; *bfpB:* Outer membrane lipoprotein. Figure produced with blast Ring Image Generator (BRIG) [52] and gradient of blast identity is shown in the legend.

PlasmidFinder revealed a large IncB/O/K/Z plasmid (86-103 kbp) in all *S. sonnei* isolates with high nucleotide similarity (96.3–99.5% identity/79-91 % coverage) to a plasmid circulating in Australian MSM from a 2 year population-level study (2016–18), AUSMDU00008333 plasmid 3 [59] (Figs 5 and S4). Compared to this 2016 reference, 598 080 and 607 307 share an AMR region harbouring resistance to aminoglycosides (*aph(6)-Id* and *aph(3′)-Ib*) and sulphonamides (*sul2*), however the two 2018 *S *. *sonnei* isolates had acquired additional tetracycline *tetR/tet(A*) genes. Notably, the Australian MSM plasmid harboured *bla*_TEM-1c_, but this element was not present in this study and the plasmid within 893 916 did not carry AMR determinants, suggesting other genes present on this plasmid are sufficient to outweigh any reproductive fitness cost.

### Analysis of IncFII plasmids and genomic context of the *bla*_CTX-M-27_ gene

pMLST revealed all four strains harboured a large IncFII ([F2:A-:B-]) plasmid (67-83kbp). They demonstrated high similarity to known plasmid pKSR100 (98–99.5% identity/80-90 % cover), which has facilitated azithromycin resistance dissemination between *Shigella* MSM lineages [15]. Since isolates were known to harbour *bla*_CTX-M-27_ they were compared to p183660, a pKSR100-like MSM plasmid which has acquired this element [16], revealing 99.7–99.9% identity/97-98 % cover. These were also compared to each other to understand the organisation and loss of respective ARGs (Fig. 4).

**Fig. 6.**
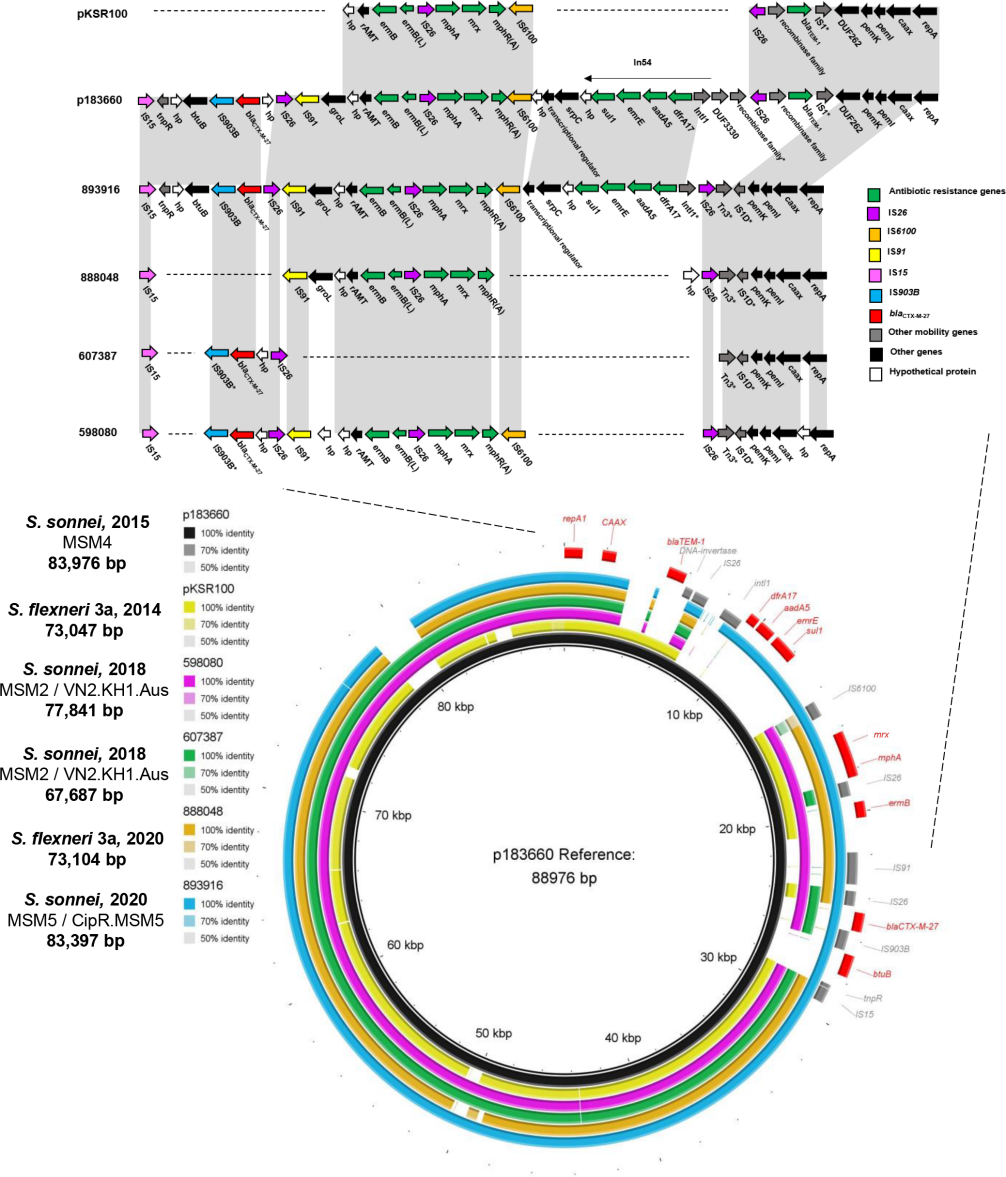
blast comparisons of IncFII pKSR100-like plasmids in four *Shigella* isolates from men who have sex with men (MSM) in England obtained with Nanopore sequencing. Three plasmids are from *S. sonnei* (isolates 598080: pink ring; 607387: green ring and 893916: blue ring) and one from *S. flexneri* 3a (888048: orange ring). The second inner ring (yellow) is pKSR100, a known MSM-related plasmid from an *S. flexneri* 3a infection and the innermost (black) ring is p183660, obtained from a man in England infected with *S. sonnei *. This has high identity to pKSR100 but had acquired the pKSR100 integron [*dfrA17, aadA5, emrE, sul1*] and a novel mobile element harbouring *bla*_CTX-M-27_. Upper: DUF- denotes domain-containing proteins. * Denotes partial features. Features are not drawn to scale. Lower: Sequences associated with mobility in this region are coloured in grey on an outer ring and antimicrobial resistance and other genes are shown on the outer ring in red. *repA1*: IncFII *repA* protein; CAAX: CPBP family intramembrane metalloprotease; *bla_TEM-1_*: β-lactamase; *intI1*: Class one integrase; *dfrA17*: Type I dihydrofolate reductase; *aadA5*: Streptomycin adenyltransferase; *emrE*: Multidrug transporter; *sul1*: Dihydropteroate synthase; *mrx:* Multidrug efflux pump; *mph(A):* Aminoglycoside phosphotransferase; *erm(B*): Dimethyladenosine transferase; *bla_CTX-M-27_*: ESBL class A β-lactamase, CTX-M-27 type; *btuB*: Vitamin B12 transporter. Figure produced with blast Ring Image Generator (BRIG) [52].

All IncFII plasmids contained a conserved ~35 kbp conjugation-associated *tra* region allowing broader host dissemination, *pemKI* toxin-antitoxin system and maintenance genes. Interestingly, ARGs were located on mosaic regions displaying unique structures for each plasmid (Fig. 6). No single ARG was found ubiquitously and no strains contained non-ESBL β-lactamase gene *bla*_TEM-1_, present in both references pKSR100 and p183660. Three-quarters of isolates harboured *erm(B*) and *mph(A*); the two 2018 *S *. *sonnei* isolates (598 080 and 607 387) had similar resistance profiles overall, differing only in loss of both genes by 607 387.

Where present, *erm(B*) was adjacent to its leading peptide (*erm(B)(L*)) and *mph(A*) to its regulatory genes, associated with IS*26* as part of an IS*26-mph(A)-mrx-mphR(A*)-IS*6100* unit, though IS6100 was not present in *S. flexneri *. Downstream of *erm(B*), these genes were always associated with a putative rRNA adenine methyltransferase, a hypothetical protein, a 60 kDa chaperonin (*groL*) and IS*91* (Fig. 6).

The *bla*_CTX-M-27_ gene (876 bp) was found on all *S. sonnei* plasmids but despite initial Illumina-based *bla*_CTX-M-27_ detection in *S. flexneri* 3a (888048), it was not observed in the assembly (Fig. 1). Mapping of trimmed Nanopore reads to a *bla*_CTX-M-27_ reference (GenBank AAO61597.1) showed it was not present in these reads (Fig. S3). As this assembly harboured a similar IncFII plasmid with *mph(A*) and *erm(B*), this suggests *bla*_CTX-M-27_ loss during storage or subculture in the 2 month duration between Illumina and Nanopore sequencing (Table S1), rather than whole plasmid loss or assembly errors (though it is possible *bla*_CTX-M-27_ was present on another plasmid that was lost during culture or through mis-assembly). Where present, *bla*_CTX-M-27_ was flanked upstream by IS*26* and downstream by IS*903B*. Isolate 893 916 was the only one to harbour the previously described pKSR100 integron present in p183660 with sulphonamide, trimethoprim and aminoglycoside resistance genes [*sul1/dfrA17/aadA5*] alongside *emrE (qacEdelta1),* quaternary ammonium compound-resistance protein. This region included heavy metal resistance protein (*srpC*) and vitamin B12 transporter (*btuB*) following *bla*_CTX-M-27_. As IS*91* is found between the pKSR100 integron and *mph(A)-erm(B*) unit in 893 916 but is lost in 888 048 which lacks this integron while harbouring *mph(A)-erm(B*), despite being present in 598 080 with the same context, it is not clear which region IS*91* is associated with (Fig. 6).

### Reorganisation of IncFII plasmids is likely driven by IS*26*

**Fig. 7.**
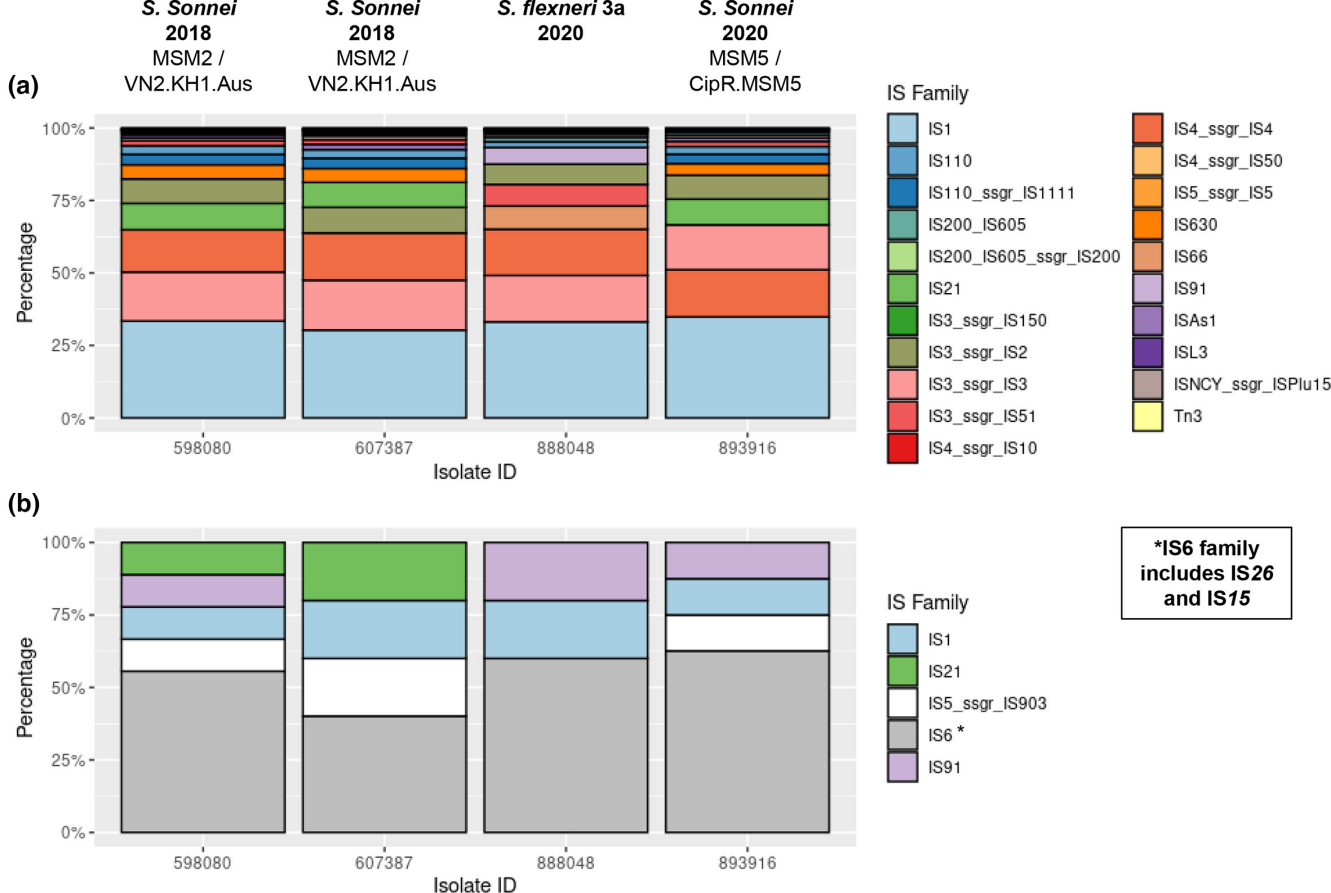
The predicted distribution of each IS family identified by ISSaga for each of the four *Shigella* isolates; *S. sonnei* (598 080, 607 387 and 893 916) and *S. flexneri* 3a (888048), by total copy number of each family. *S. sonnei* lineage is shown below isolation year where applicable. (Note: in some cases there are >1 different IS per family). (**a**) Distribution of each family within contigs identified as chromosomal sequences for each isolate. (**b**) Distribution of each IS family within the IncFII pKSR100-like plasmid present within each isolate.

IS elements drive genomic evolution and typically comprise a transposase, catalysing enzymatic mobility, with flanking short terminal inverted repeats (TIRs) (transposase recognition sites) [60]. ISsaga revealed 577, 520 and 588 total predicted IS in *S. sonnei* genomes (598080, 607387 and 893 916 respectively) and 504 in *S. flexneri* 3a (Table 2); lower copy numbers in 607 387 are likely due to breaks between contigs. The most frequently identified IS family was IS*1*, accounting for 28.4, 26.3 and 30.8% in *S. sonnei* and 28.9% in *S. flexneri *. Chromosomal IS family proportions are similar between strains (Fig. 7a) but only *S. flexneri* harbours IS*66* and remarkably, no copies of the IS*6* family are present chromosomally in any isolates, yet dominate on pKSR100-like plasmids; mainly IS*26* (Fig. 7b).

IS*26* and IS*15* were always flanked by known 14 bp TIR (5′-GGCACTGTTGCAAA-3′) [61], suggesting possible recent acquisition without amelioration. When IS*26* integrates, in some circumstances this results in 8 bp target sequence duplication (TSD) and flanking TIRs. No TSDs were manually identified so further study is needed to understand translocation mechanisms and acquisition dynamics. IS concentration on these plasmids and flanking of lost regions by IS suggests possible IS-mediated deletion and microevolution driven by IS insertion into the plasmid backbone.

## Discussion

Overall our data show long-read sequencing supports elucidation of genomic AMR context among MSM-associated *Shigella* strains. This includes ESBL (*bla*_CTX-M-27_), borne on a mosaic ARG region within IncFII plasmids which are likely to have been reorganised by IS*26*. This, together with their phylogenetic position within MSM clades, can aid understanding of how AMR is tied to persistence in the population.

Combinations of mutations and presence of ARGs within isolates in this study promote resistance to all current first-line antimicrobials (macrolides, fluoroquinolones and third-generation cephalosporins) [62]. *S. sonnei* isolates harboured Tn7/Int2; this facilitated worldwide dissemination of global lineage III by allowing adaptation to multiple antibiotics in the environment [11]. Only the *S. flexneri* isolate harboured the SRL-MDRE with *bla*_OXA-1_ conferring ampicillin resistance, congruent with an Iranian study which suggested *S. flexneri bla*_OXA-1_ host preference [63]. The *S. flexneri* isolate harboured secretion autotransporter toxin *sat* and *sepA* which is associated with intense abdominal pain [64]. However, *S. sonnei* isolates exhibited higher numbers of virulence genes, including *senB* and *sigA* associated with bloody diarrhoea and fever respectively [65]. Presence of isolates with high levels of both AMR and virulence factors could be a consequence of clinically severe cases requiring antibiotics and exerting selective pressure and are a public health concern due to the challenge this poses to outbreak management.

Reduced susceptibility to azithromycin (RSA) in *Shigella* was not present in England in 2002 [66], but has arisen possibly due to pressure from treatment of other STIs in MSM [67] and triple QRDR mutations promoted emergence of fluoroquinolone-resistant *S. sonnei* in South Asia which later transmitted internationally [14]. ESBL prevalence is increasing, especially in Asia, North America and Europe [68]; WGS of 335 *S *. *sonnei* isolates associated with domestic acquisition in England and Wales (2015–16) denotes CTX-M genes in 12% of isolates and *bla*_CTX-M-27_ in 2.7% of those, though CTX-M-15 was most common [69]. Use of ceftriaxone to treat shigellosis should be carefully considered, especially in areas with RSA, due to possible co-selection of ESBL with macrolide resistance genes on the same plasmid [15, 59]. There are risks that ESBL-producing strains may spread among HIV-positive MSM with increased biologically susceptibility; use of networking applications is a transmission risk factor [70] so could be utilised for awareness campaigns and contact tracing, as shown effectively during a Berlin MSM hepatitis A outbreak [71].

In this study, *aph(6)-Id/aph(3)-Ib*/*sul2/tet(A*) carriage was on a large (~103 kbp) IncB/O/K/Z plasmid with Australian links. *bla*_CTX-M-27_-positive *S. sonnei* has recently driven a prolonged MSM outbreak in Australia [17], with one successful sublineage harbouring an AMR profile consistent with one isolate in this study, 598 080 (*mphA, ermB, dfrA1* and *sul2* genes with *gyrA* S83L). Typing of *S. sonnei* isolates 598 080 and 607 387 determined them as VN2.KH1.Aus; Australian isolates emerging from Kahnh Hoa Subclone 1 [47]. Though not designated as MSM-linked by this scheme, this could represent travel-associated introduction into England and subsequent MSM transmission.

We found *bla*_CTX-M-27_ was present on IncFII plasmids with high identity to a known plasmid linked to an English MDR *S. sonnei* cluster, p183660 (which harbours *bla*_CTX-M-27_ and has high identity to pKSR100) [15]. We observed *bla*_CTX-M-27_ displace *bla*_TEM-1_, and *bla*_CTX-M-27_ loss between sequencing rounds was observed in *S. flexneri* 3a. This gene possibly has lower stability in this species; none of the 49 other *S. flexneri* isolates harboured *bla*_CTX-M-27_ and globally *bla*_CTX-M-27_ seems relatively rare in *S. flexneri* [72]. Alternatively, as this isolate also harboured chromosomal β-lactamases *ampC* and *bla*_OXA-1_, IS activity may have led to rapid plasmid *bla*_CTX-M-27_ loss in the absence of antimicrobial pressure. It is interesting that isolate 607 387 lost both *mphA* and *ermB* (two macrolide resistance mechanisms; phosphotransferase-mediated modification and methylase ribosome target-site modification) together with two MSM clade 2 members, whereas many *S. sonnei* isolates in non-MSM clades harboured only *mph(A*) (no isolate contained *erm(B*) alone).

Insertion sequences are challenging to identify with short-read data but facilitate ongoing species diversification in *Shigella* through gene interruption, deletion and genome reorganisation with convergence on streamlined genomes [73]. Hawkey *et al*. showed expansion of IS*1*, IS*2*, IS*4*, IS*600* and IS*911* in Shigella genomes; similarly, we showed high proportions of IS*1*, IS*2* and IS*4* with differing *S. sonnei* and *S. flexneri* IS profiles. IS activity causes potentially deleterious outcomes (e.g. ARG loss) but allows flexibility to create diverse structures; some of which are likely to be selected for [74]. It has been suggested if insertion of IS*26* causes no deleterious effects it acts as a ‘founder element’ where further IS*26* insertion occurs preferentially next to another [75], enabling rapid novel ARG acquisition. Concordantly, here IS*26* preferentially inserted into IncFII plasmids and IS*15* was present close to *bla*_CTX-M-27_, corresponding to one IS*26* copy inserted into another. Similar to our genetic platform, *bla*_CTX-M-27_ was flanked by IS*26* and IS*903B* in *E. coli* ST38 IncF plasmids [76], suggesting a possible CTX-M reservoir. Currently annotated *bla*_CTX-M-27_-carrying *Shigella* plasmids are limited to *S. sonnei* p183660 (GenBank KX008967.1) and Swiss pEC732_2 (GenBank CP015140.1). Within the latter a contrasting IS*26*-IS*903B*Δ-*bla*_CTX-M-27_-IS*Ecp1*Δ-IS*26* unit was identified [19], though it is possible IS26 was inserted into IS*Ecp1* (promoting high-level expression) in our study but not identified.

Although the mechanistic basis of *bla*_CTX-M-27_ and *mph(A)-erm(B)* loss is speculative, this could reflect intramolecular IS*26* transposition in cis, resulting in deletion of DNA between the IS and target site, leaving one IS copy [77]. IS*26* is known to generate 8 bp target site duplications during cointegrate formation [77]; investigating these within additional ESBL plasmids over time would provide insights regarding temporal and spatial dynamics of AMR acquisition and loss.

This study is limited in low isolate numbers from a single region without patient exposure data or investigation of other CTX-M genes in additional isolates in PHE's collection; we cannot extrapolate ARG patterns and their position within MGEs in MSM-related strains over time or characterise risk factors. In *S. sonnei* there is generally high concordance between WGS ARG presence and phenotype when 100% read coverage and >90 % nucleotide identity thresholds are used [69], though phenotypic data did not correlate with genotype for tetracycline resistance in 893 916 and the underlying mechanism was not investigated. If long-read sequencing becomes viable on a routine scale, this would significantly aid plasmid monitoring to predict AMR dissemination, especially as *bla*_CTX-M-27_ seems to have been acquired on multiple occasions.

## Conclusions

*Shigella* spp. are concerning due to their extraordinary ability to acquire and disseminate AMR. As ESBL genes can be propagated by movement on small mobile genetic elements, dissemination of ‘epidemic’ plasmids and via clonal spread, WGS surveillance of all three avenues with international data sharing is necessary to inform coordinated and responsible antimicrobial strategies.

## Supplementary Data

Supplementary material 1
