# Supplementary material for: Acquisition and loss of CTX-M plasmids in Shigella species associated with MSM transmission in the UK
Source: Microb Genom. 2021 Aug 24;7(8):000644. doi: 10.1099/mgen.0.000644 (PMC8549364; doi:10.1099/mgen.0.000644)
Supplement: Supplementary material 1 [file mgen-7-0644-s001.pdf]

Supplementary Information

Acquisition and loss of CTX-M plasmids in *Shigella* species associated with MSM transmission in the UK

Rebecca K. Locke[1], David R. Greig[2,3], Claire Jenkins[2], Tim J. Dallman[2,3] and Lauren A. Cowley[1]

[1]University of Bath, Claverton Down Campus, Bath, United Kingdom
[2]Gastrointestinal Reference Services, Public Health England, London, United Kingdom
[3]Division of Infection and Immunity, The Roslin Institute and Royal (Dick) School of Veterinary Studies, University of Edinburgh, Easter Bush, EH25 9RG, United Kingdom

Correspondence to (E-mail): rkl30@bath.ac.uk

Keywords: *Shigella*, MSM, Antimicrobial Resistance, ESBL, CTX-M, Public Health

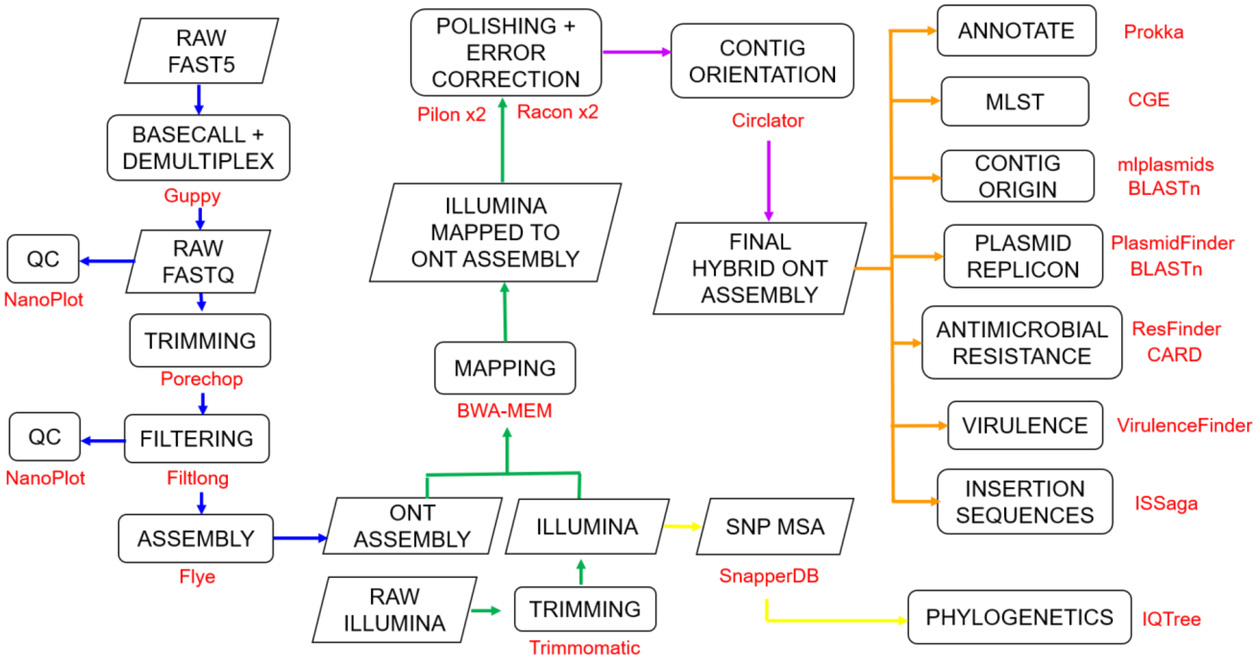

Fig S1. Flowchart of the bioinformatic pipeline used to investigate and characterise the antimicrobial resistance determinants of four *Shigella* strains sequenced with Nanopore and Illumina technologies. Detailed steps and parameters are provided in the main article. Steps are as follows; 1) assembly of long-reads (blue); 2) polishing of the long-read assembly using Illumina data (green); 3) contig orientation (fix start position) (purple); 4) detailed investigation of resistance determinants, whole genome MLST and plasmid MLST, insertion sequence families and virulence factors present (orange) and 5) phylogenetic analysis using a SNP multiple sequence alignment (MSA) for *S. sonnei* and *S. flexneri* separately with representatives from PHE's collection to provide context (yellow).

Table S1. Dates of sequencing for the *Shigella* isolates. Note the timeframe between Illumina and Oxford Nanopore Technologies (ONT) sequencing for isolate 888048 was two months, during which it is hypothesised that this isolate lost *bla*$_{CTX-M-27}$ from a plasmid.

Isolate ID	Illumina sequencing date	Nanopore sequencing date
**598080**	05.09.2018	05.03.2019
**607387**	20.09.2018	05.03.2019
**888048**	15.02.2020	01.04.2020
**893916**	26.02.2020	01.04.2020 and 15.04.2020 (2 runs)

Table S2. Quality assessment of Nanopore long-reads after Filtlong filtering to 30x theoretical coverage, as determined by NanoPlot. These reads were used to assemble with Flye and Unicycler.

Isolate ID	No. of reads	Total bases	Mean read length	Mean read quality	Read length N_{50}
**598080**	10,286	141,003,424	13,708.3	9.0	14,296
**607387**	39,526	105,168,279	2,660.7	8.8	3,194
**888048**	11,181	141,000,982	12,610.8	10.5	16,480
**893916**	3,204	141,007,921	44,010.0	10.2	43,791

Table S3. Comparison between the genomes assembled with Flye (long-read only assembly with Illumina read polishing), and Unicycler (using Illumina reads as a hybrid assembly approach). Flye assemblies were polished with Ilumina reads with two rounds each of Pilon and Racon. Unicycler assemblies were polished with Racon twice (as multiple rounds of Pilon polishing is an in-built step). Assembly statistics were determined with QUAST (Gurevich et al., 2013).

	**Isolate ID**			
	**598080**	**607387**	**888048**	**893916**
	**Flye v2.7.1**			
**Contigs**	6	37	5	4
**Total bases**	5,282,682	5,315,865	4,839,032	5,196,904
**N_{50}**	4,879,016	897,518	4,519,004	4,813,904
	**Unicycler v0.4.8**			
**Contigs**	27	71	27	35
**Total bases**	5,335,473	5,190,829	4,846,214	5,277,071
**N_{50}**	4,246,387	577,034	3,881,855	4,815,305

Table S4. Detailed assembly information for the four *Shigella* isolates. Contigs highlighted in grey are investigated in detail in this study.

A) ***S. sonnei* isolate 598080, deposited in GenBank under the accession JAENSM000000000.**

Contig	Replicon	Inc type	Size (bp)	GC content (%)	Notes: AMR and virulence factors
**1**	Chromosome	-	4,877,015	51.00	**AMR**: Tn7/Int2 integron containing *dfrA1, sat2, aadA1* Also harbours *mdf(A).* **Virulence**: *gad, ipaH*9.8, *iucC, iutA, lpfA, senB, sigA, sitA, terC.*
**2**	Chromosome	-	21,621	45.94	
**3**	Plasmid 1	IncFII [F2:A-:B-]	77,841	52.22	**AMR:** 98% cover, 99.93% identity to p183660 (KX008967.1). AMR: harbours *mph(A), erm(B)* and *bla*$_{CTX\text{-}M\text{-}27}$
**4**	Plasmid 2	IncB/O/K/Z	103,000	54.09	**AMR:** 99.46% identity, 79% cover to pAUSMDU00008333_3 (LR213460.1). Harbours *aph(6)-Id, aph(3")-Ib, sul2* and *tet(A).*
**5**	Plasmid 3	IncFII [F27:A-:B-]	194,330	45.68	**Virulence** plasmid. Harbours *ipaD* and *virF.*
**6**	Likely plasmid		6774	50.68	**Virulence:** 100% cover, 100% identity to pAUSMDU00010534_4. Virulence: harbours *celb.*

B) *S. sonnei* isolate 607387. Deposited in GenBank under accession JAEMEC000000000. Contigs were rearranged according to the high quality *S. sonnei* 53G reference (GenBank accession NC_016822.1).

Contig	Replicon	Inc type	Size (bp)	GC content (%)	Notes: AMR and virulence factors
**1**	Chromosome	-	2,500,534	51.33	**AMR**: Tn7/Int2 integron containing *dfrA1, sat2, aadA1.*<br>Virulence: *gad, iutC, iutA, lpfA, terC.*
**2**	Chromosome	-	6972	50.75	
**3**	Chromosome	-	178,711	51.69	
**4**	Chromosome	-	239,447	50.83	**AMR**: harbours *mdf(A).*
**5**	Chromosome	-	4069	51.93	
**6**	Chromosome	-	2933	47.12	
**7**	Chromosome	-	146,944	50.20	
**8**	Chromosome	-	99,559	49.05	
**9**	Chromosome	-	22,458	46.43	
**10**	Chromosome	-	16,644	49.68	
**11**	Chromosome	-	171,501	49.91	
**12**	Chromosome	-	136,782	50.20	
**13**	Chromosome	-	10,758	42.97	
**14**	Chromosome	-	261,218	50.54	**Virulence**: harbours *sitA* and *gad.*
**15**	Chromosome	-	7566	46.97	
**16**	Chromosome	-	897,518	50.97	**Virulence**: harbours *senB*.
**17**	Chromosome	-	159,310	51.63	
**18**	Chromosome	-	2162	52.54	
**19**	Chromosome	-	20,947	52.53	
**20**	Chromosome	-	3394	45.64	**Virulence**: harbours *lpfA.*
**21**	Chromosome	-	4322	35.33	
**22**	Chromosome	-	5277	45.65	
**23**	Chromosome	-	3769	42.16	
**24**	Chromosome	-	2690	46.06	
**25**	Plasmid 1	IncFII [F27:A-:B-]	182,482	45.18	**Virulence** plasmid pINV. Harbours *ipaD* and *virF.*
**26**	Likely plasmid		4554	48.18	
**27**	Likely plasmid		3718	51.53	
**28**	Plasmid 2	IncFII [F2:A-:B-]	67,687	51.65	**AMR:** 99.76% identity, 97% cover to p183660 (KX008967.1). AMR: Harbours $bla_{CTX\text{-}M\text{-}27.}$
**29**	Likely plasmid		2689	46.26	99.96% identity to *S. sonnei* pAUSMDU00010534_08 (CP45940.1).
**30**	Plasmid 3	Col(BS512)	2160	47.36	

**31**	Likely plasmid		13,543	50.68	**Virulence**: Harbours *celb* endonuclease colicin E2
**32**	Likely plasmid		10,435	48.55	99.92% identity to *S. sonnei* pAUSMDU000100534_05.
**33**	Likely plasmid		4275	54.11	
**34**	Likely plasmid		2198	51.14	
**35**	Plasmid 4	IncB/O/K/Z	103,015	54.09	99.46% identity, 79% cover to *S. sonnei* pAUSMDU00008333_3 (LR213460.1). Harbours *aph(6)-Id, aph(3")-Ib, sul2* and *tet(A).*
**36**	Likely plasmid		5166	47.44	99.09% identity to *E. coli* p2NQ3 (CPO24722.1).
**37**	Likely plasmid		8458	40.71	99.03% identity to *S. sonnei* pCFSAN030807_7 (CPO23652.1) with type IV secretion system protein VirB5.

C) *S. flexneri* 3a isolate 888048. Sequences are deposited in GenBank separately and their accession numbers are listed below.

**Contig**	**Replicon**	**Inc type**	**Size (bp)**	**GC content (%)**	**Notes: AMR and virulence factors**	**Accession**
**1**	Chromosome	-	4,519,004	50.91	**AMR**: SRL-MDRE containing *aadA1*, *bla*$_{OXA\text{-}1}$, *catA1, tet(B).* Also harbours *mdf(A).* **Virulence**: *gad, ipaH*9.8, *iucC, iutA, sitA, terC.*	CP066809
**2**	Plasmid 1	IncFII [F2:A-:B-]	73,104	52.24	**AMR:** 98% cover, 99.97% identity to p183660 (KX008967.1). Harbours *mph(A)* and *erm(B)*	MW396860
**3**	Plasmid 2	IncFII [F27:A-:B-]	231,092	46.07	**Virulence** plasmid pINV. Harbours *ipaD, sepA* and *virF.*	MW396862
**4**	Likely plasmid		10,115	47.15	61% cover, 97.52% identity to *S. flexneri*	MW396863

					pFDAARGOS_535 (CP034063.1).	
**5**	Likely plasmid		5,717	42.33	100% cover, 99.98% identity to pRHBSTW-00822_4 (CP056318.1).	MW396861

D) *S. sonnei* isolate 893916. Sequences are deposited in GenBank separately and their accession numbers are listed below.

**Contig**	**Replicon**	**Inc type**	**Size (bp)**	**GC content (%)**	**Notes and AMR**	**Accession**
**1**	Chromosome	-	4,813,904	51.03	**AMR**: Tn7/Int2 integron containing *dfrA1 and aadA1.* **Virulence**: *gad, ipaH*9.8, *iucC, iutA, lpfA, senB, sigA, sitA, terC.*	CP066810
**2**	Plasmid 1	IncFII [F2:A-:B-]	83,397	52.52	**AMR:** 98% cover, 99.97% identity to p183660 (KX008967.1). AMR: Harbours *dfrA17, aadA5, emrE, sul1, mph(A), erm(B) and* $bla_{CTX\text{-}M\text{-}27}$.	MW396858
**3**	Plasmid 2	IncFII [F27:A-:B-]	212,787	45.20	**Virulence** plasmid pINV. Harbours *ipaD* and *virF*.	MW396859
**4**	Plasmid 3	IncB/O/K/Z	86,816	52.95	**AMR:** 96.26% identity and 91% query cover to *S. sonnei* pAUSMDU00008333_3 (LR213460.1), does not harbour resistance determinants.	MW396864

Table S5. Known mobile genetic elements found in MSM in *Shigella.* Reproduced with slight adaptations from (Baker et al., 2018).

**Type**	**Mobile genetic element**	**Genes**	**Resistance conferred**
**Chromosomal Island**	**SRL-MDRE**	bla_{OXA-1}	Ampicillin
		catA1	Chloramphenicol
		aadA1	Aminoglycosides
		tet(B)	Tetracyclines
	**Tn7/Int2**	*aadA1*	Aminoglycosides
		sat2	Streptothricin
		dfrA1	Trimethoprim
**Plasmid**	**pKSR100 (conjugative R-plasmid)**	*erm*(B)	Macrolides (erythromycin)
		mph(A)	Macrolides (azithromycin)
		bla_{TEM-1}*	Ampicillin
	**pKSR100 integron**	*dfrA17*	Trimethoprim
		aadA5	Aminoglycosides
		sul1	Sulphonamides
	**pCERC-1 (R-plasmid) / spA**	*dfrA14**	Trimethoprim
		sul2	Sulphonamides
		aph(3")-Ib (strA)	Aminglycosides
		aph(6)-Id (strB)	Aminoglycosides

*Note: bla_{TEM-1} and *dfrA14* were not found in the four strains sequenced in this study.

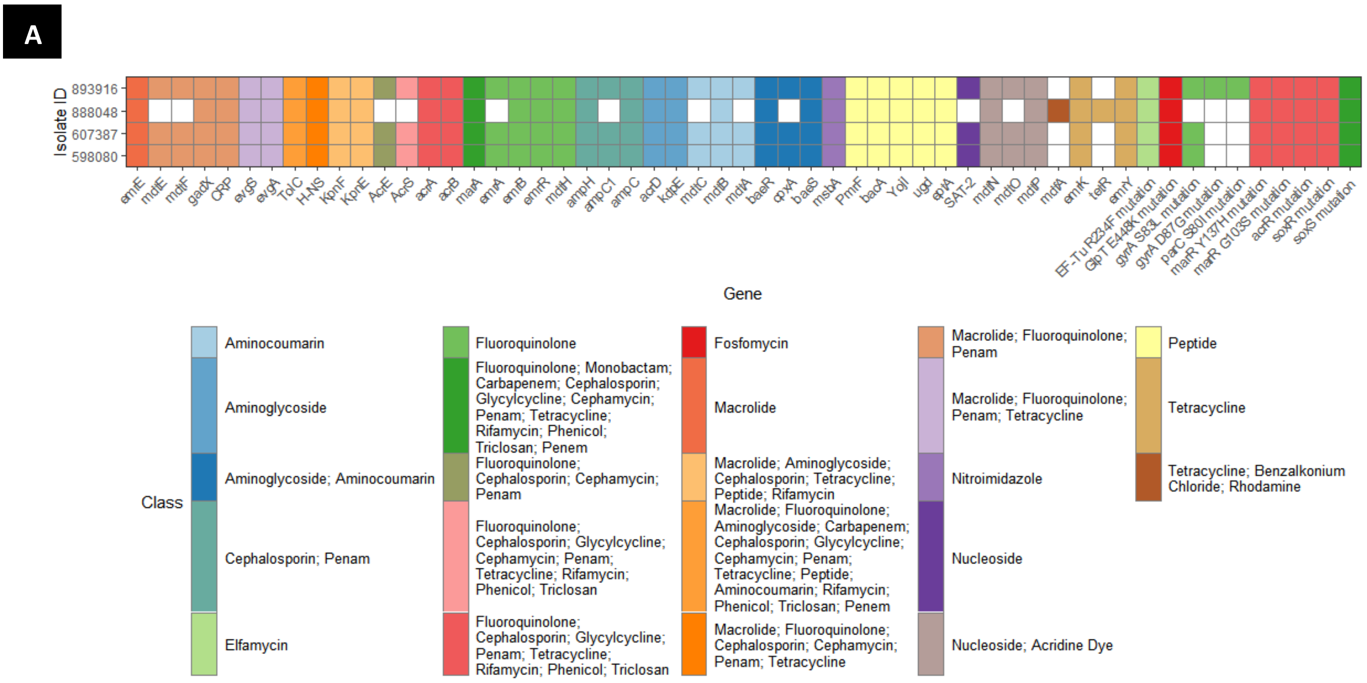

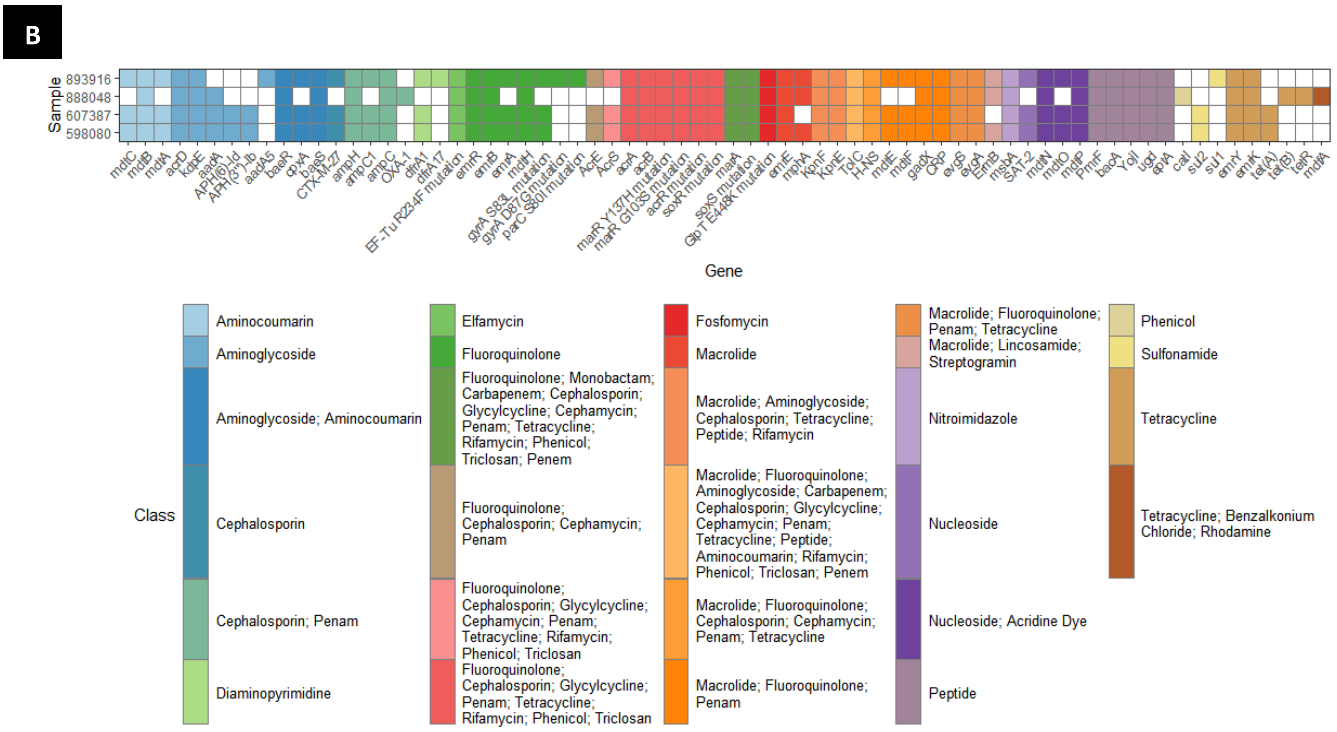

Fig. S2. A) Antimicrobial resistance genes and chromosomal mutations identified by the Comprehensive Antibiotic Resistance Database (CARD) Resistance Gene Identifier tool. Perfect and Strict hits are included, where variants are likely still functional. Genes found by ResFinder shown in Fig. 1 are omitted. **B) All resistance genes identified by CARD (Perfect & Strict hits).** All hits are shown, including those also found by ResFinder.

A

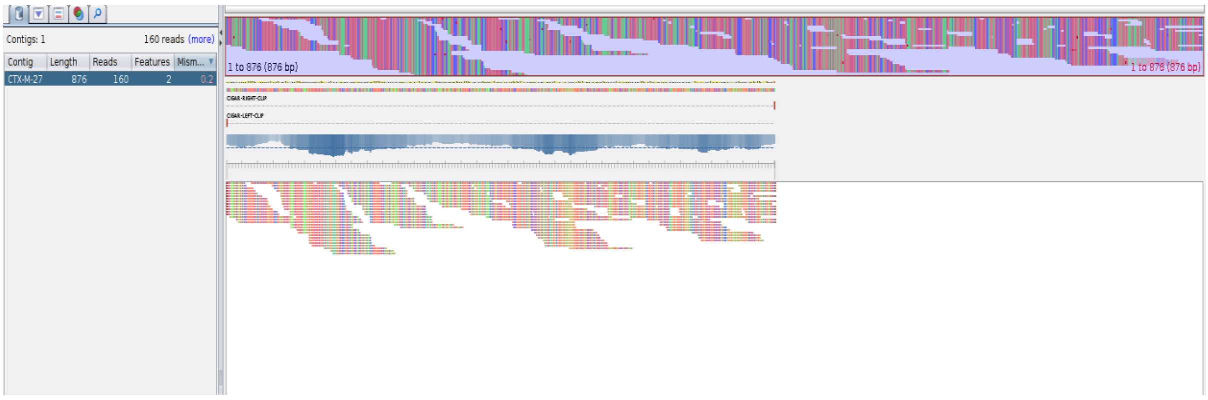

B

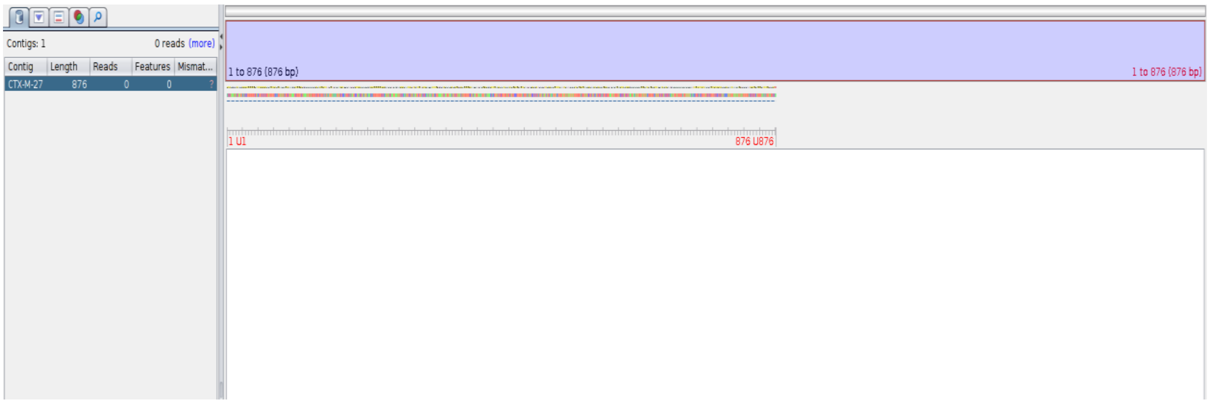

Fig. S3. Reads mapped to *bla*$_{\text{CTX-M-27}}$ for *S. flexneri* 3a isolate 888048. (A) 160 short-read Illumina sequences mapped to the *bla*$_{\text{CTX-M-27}}$ gene (Accession Number AAO61597.1) with BWA-MEM. **(B)** Empty BAM file showing no Nanopore long-reads mapped to the *bla*$_{\text{CTX-M-27}}$ gene with minimap2. This isolate was sequenced using Oxford Nanopore technologies around two months after Illumina sequencing, so it is likely that this sample had lost *bla*$_{\text{CTX-M-27}}$ between each round. BAM file is visualised using Tablet (Milne et al., 2009).

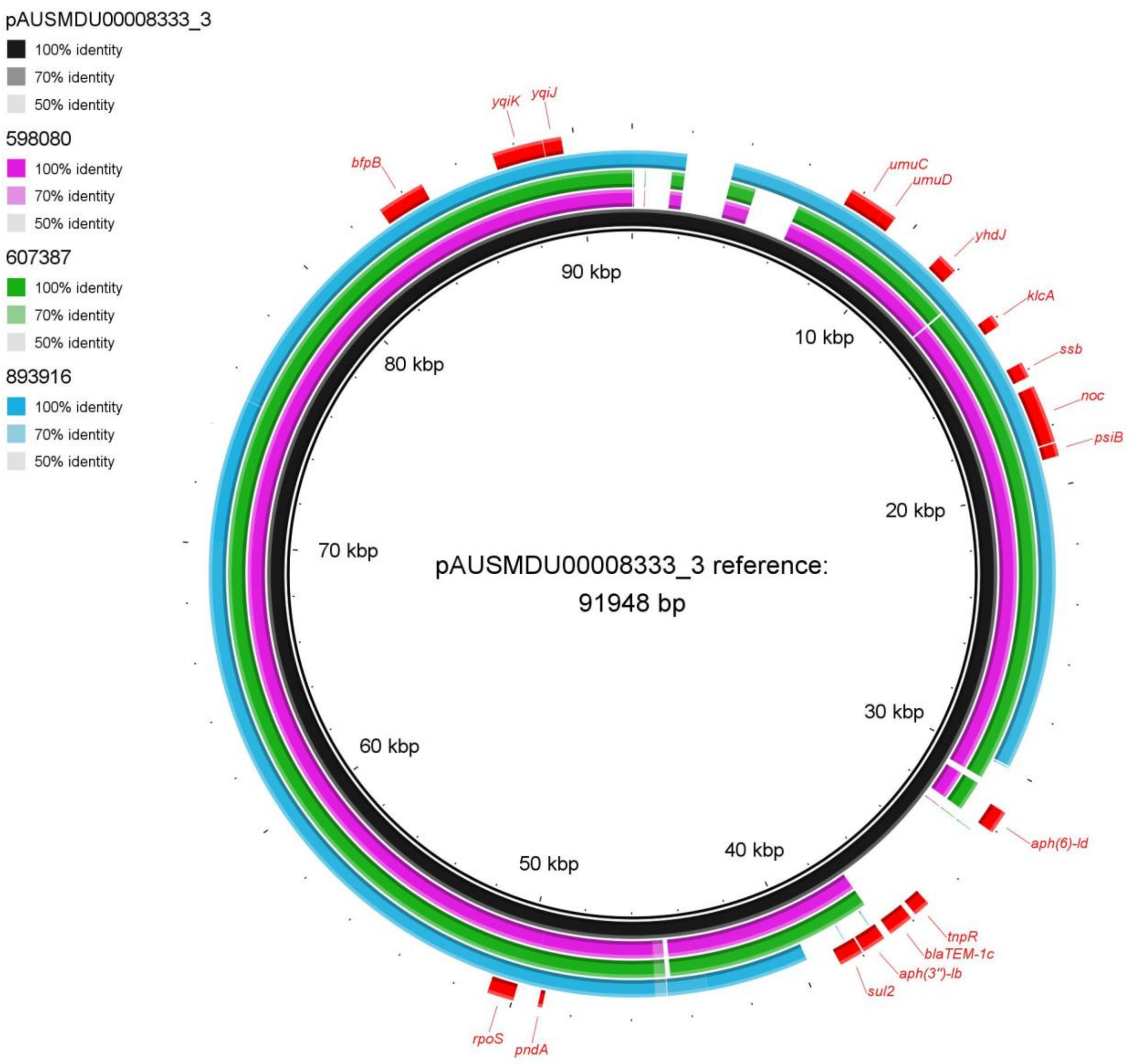

Fig S4. Genomic comparison of three *S. sonnei* IncB/O/K/Z plasmids from this study generated with long Nanopore reads with pAUSMDU00008333_3 (GenBank LR213460.1), a plasmid known to be circulating in Australian MSM, as reference. This is similar to Fig. 5 but shows that plasmids in these three strains have lost $bla_{\text{TEM-1c}}$ and *tnpR* compared to the plasmid isolated from the Australian strain. Figure produced with BLAST Ring Image Generator (BRIG) (Alikhan et al., 2011).

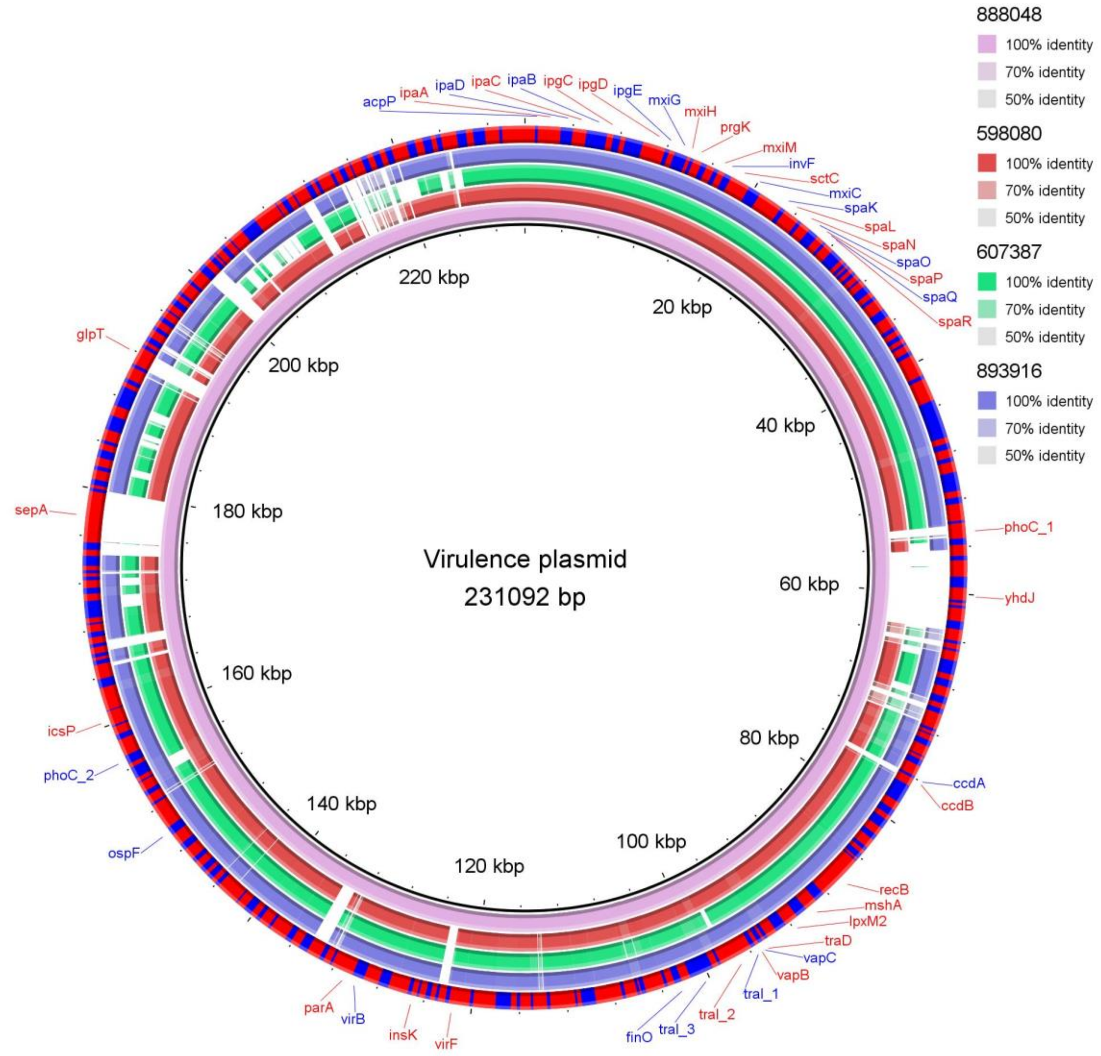

Fig. S5. pINV >200kbp virulence plasmid present in all four *Shigella* isolates associated with men who have sex with men in England. Plasmids were constructed by assembly of Nanopore reads with Flye. Virulence plasmid from *S. flexneri* 3a isolate 888048 (inner ring, pink) is used as a reference and for annotation (as it is the largest). Second ring (red) is virulence plasmid from *S. sonnei* isolate 598080, third ring (green) is virulence plasmid from *S. sonnei* isolate 607387 and fourth (blue) is virulence plasmid from *S. sonnei* isolate 893916. Figure produced with BLAST Ring Image Generator (Alikhan et al., 2011).

Table S6. pMLST results from whole genome assemblies with PubMLST (Jolley et al., 2018)**.**

**Isolate ID**	**pMLST**
**598080**	[F2:A-:B-], [F27:A-:B-], *ardA*_28 from IncI1 scheme (unknown ST)
**607387**	[F2:A-:B-], [F27:A-:B-], *ardA*_28 from IncI1 scheme (unknown ST)
**888048**	[F2:A-:B-], [F27:A-:B-]
**893916**	[F2:A-:B-], [F27:A-:B-], *ardA*_2 from IncI1 scheme (unknown ST)

References

Alikhan, N.F., Petty, N.K., Ben Zakour, N.L. and Beatson, S.A., 2011. BLAST Ring Image Generator (BRIG): Simple prokaryote genome comparisons. *BMC Genomics*, 12(1), p.402. https://doi.org/10.1186/1471-2164-12-402.

Baker, K.S., Dallman, T.J., Field, N., Childs, T., Mitchell, H., Day, M., Weill, F.X., Lefèvre, S., Tourdjman, M., Hughes, G., Jenkins, C. and Thomson, N., 2018. Genomic epidemiology of Shigella in the United Kingdom shows transmission of pathogen sublineages and determinants of antimicrobial resistance. *Scientific Reports*, 8(1). https://doi.org/10.1038/s41598-018-25764-3.

Gurevich, A., Saveliev, V., Vyahhi, N. and Tesler, G., 2013. QUAST: Quality assessment tool for genome assemblies. *Bioinformatics*, 29(8), pp.1072–1075. https://doi.org/10.1093/bioinformatics/btt086.

Jolley, K.A., Bray, J.E. and Maiden, M.C.J., 2018. Open-access bacterial population genomics: BIGSdb software, the PubMLST.org website and their applications. *Wellcome Open Research*, 3. https://doi.org/10.12688/wellcomeopenres.14826.1.

Milne, I., Bayer, M., Cardle, L., Shaw, P., Stephen, G., Wright, F. and Marshall, D., 2009. Tablet-next generation sequence assembly visualization. *Bioinformatics*, 26(3), pp.401–402. https://doi.org/10.1093/bioinformatics/btp666.
